# Fine-scale species delimitation: speciation in process and periodic patterns in nudibranch diversity

**DOI:** 10.3897/zookeys.917.47444

**Published:** 2020-03-09

**Authors:** Tatiana Korshunova, Klas Malmberg, Jakov Prkić, Alen Petani, Karin Fletcher, Kennet Lundin, Alexander Martynov

**Affiliations:** 1 Koltzov Institute of Developmental Biology RAS, 26 Vavilova Str., 119334 Moscow, Russia Koltzov Institute of Developmental Biology RAS Moscow Russia; 2 Zoological Museum, Moscow State University, Bolshaya Nikitskaya Str. 6, 125009 Moscow, Russia Moscow State University Moscow Russia; 3 Aquatilis, Nostravägen 11, S-41743, Gothenburg, Sweden Aquatilis Gothenburgh Sweden; 4 Getaldiceva 11, C 21000 Split, Croatia Unaffiliated Split Croatia; 5 Put Kotlara 6, C 23000 Zadar, Croatia Unaffiliated Zadar Croatia; 6 Port Orchard, Washington, 98366, USA Unaffiliated Port Orchard United States of America; 7 Gothenburg Natural History Museum, Box 7283, SE-40235, Gothenburg, Sweden Gothenburg Natural History Museum Gothenburg Sweden; 8 Gothenburg Global Biodiversity Centre, Box 461, SE-40530, Gothenburg, Sweden Gothenburg Global Biodiversity Centre Gothenburg Sweden

**Keywords:** biodiversity, biological periodicity, multilevel organism diversity, phylogeny, speciation, species problem

## Abstract

Using the nudibranch genus *Amphorina* as a model, ongoing speciation is demonstrated, as well as how periodic-like patterns in colouration can be included in an integrated method of fine-scale species delimitation. By combining several methods, including BPP analysis and the study of molecular, morphological, and ecological data from a large number of specimens within a broad geographic range from northern Europe to the Mediterranean, five species are recognised within the genus *Amphorina*, reviewed here for the first time. Two new species from the southwestern coast of Sweden are described, *A. viriola***sp. nov.** and *A. andra***sp. nov.** Evidence is provided of a recent speciation process between the two closely related, yet separate, species which inhabit the same geographic localities but demonstrate strict water depth differentiation, with one species inhabiting the shallow brackish top layer above the halocline and the other species inhabiting the underlying saltier water. The results presented here are of relevance for currently debated issues such as conservation in relation to speciation, fine species delimitation, and integration of molecular, morphological and ecological information in biodiversity studies. The periodic approach to biological taxonomy has considerable practical potential for various organismal groups.

## Introduction

Species delimitation, and hence the degree of separation between different groups of biological organisms, is a pivotal concept for modern biology, despite the fact that there is no universal agreement about the species concept itself (Stanton et al. 2019). The universal species concept proposed by de Queiroz (2007) – i.e., that species represent separately evolving evolutionary lines without any other defining characters – potentially implies the impossibility of taxonomically defining characters at a general scale. It also makes species delimitation a significant modern problem because of the considerable proportion of hidden diversity that is often seemingly impossible to detect by morphological examination alone. Thus, a majority of modern approaches imply that the addition of molecular methods to traditional morphology-based taxonomy is necessary for species identification (Nylander et al. 2004; Puillandre et al. 2012; Yang 2015; Sukumaran and Knowles 2017). In any outcome there are numerous discrepancies between species as a taxonomic unit and the underlying natural phenomenon (Callahan et al 2017; Zachos 2018a). For species as a systematic unit we only need to represent a firm taxonomic diagnosis (Winston 1999), whereas underlying natural phenomena may be represented by multilevel organism diversity fuelled by a dynamic evolutionary process (Korshunova et al. 2019a) in a species-population complex continuum (Coates et al. 2018). Therefore, it is quite common that when taxonomists come across morphologically difficult-to-distinguish species complexes at different levels of evolutionary differentiation they commonly simplify the underlying organism processes in order to taxonomically present an apparently “well-enough-delineated” species. Difficulties in assessing morphological distinctions, the apparent ease of species recognition through molecular analyses, and underestimation of the actual complex genetic and epigenetic processes within the ontogenetic framework of any organism often result in statements about the impossibility of finding reliable morphological diagnostic differences in many recently described species, thereby commonly denouncing them as cryptic (Singhal et al. 2018; Nygren et al. 2018; Bannikova et al. 2019; Struck et al. 2018). Therefore, developing approaches that will help reveal the multilevel nature of organism diversity is highly desirable, since that would place the issue within a more complex framework than traditional strictly hierarchical and diagnosis-based taxonomy.

Here we are using a complex case of nudibranch mollusc species of the genus *Amphorina* (family Eubranchidae), which are externally very difficult to distinguish as a suitable example to show the limits of currently prevailing species diagnostic methods. To delimit several closely related and similar-looking European species of this genus, we applied a suite of methods, including molecular phylogenetic analysis, BPP and ABGD, to show that several molecular clades contain all possible varieties of external morphological characters within the same species. This makes species identification and delimitation by external morphological characters apparently difficult and thus, at a first glance, calls for the existence of cryptic species. However, subsequent analysis of the colour variation within each species shows that the diversity is not fully random but can be arranged in periodic-like rows for each species. Periodic patterns in the formation of morphological diversity were reliably estimated theoretically (Hess 2000; Hiscock and Megason 2015) and have most recently been confirmed, with robust developmental data, from different vertebrates such as fishes, birds, and mammals (Haupaix et al. 2019). However, a practical application of the periodic approach in biological taxonomy is extremely rare, although there are a few promising studies on the application of periodic patterns in proteins (Taylor 2002) and in the phylotypic ontogenetic stages of higher-level taxonomic categories (Martynov and Korshunova 2015). Therefore, we show that the combination of molecular methods with a periodic morphological approach plus ecological data facilitates species delimitation and allows the discovery of fine diagnostic characters even in externally difficult to distinguish and highly similar taxonomic complexes.

## Materials and methods

Material for this study was obtained by scuba diving at widely separate locations in Europe: in the, Croatia, France, Norway, Sweden, Spain, and the United Kingdom. The specimens were deposited in the Gothenburg Natural History Museum (**GNM**) and in the Zoological Museum of Lomonosov Moscow State University (**ZMMU**). Integration of molecular and morphological data as well as phylogenetic and biogeographical patterns were used. The external and internal morphology of specimens was studied using digital cameras, under a stereomicroscope and with a scanning electron microscope.

Specimens of *Amphorina* were sequenced in Gothenburg and in Moscow for the mitochondrial genes cytochrome c oxidase subunit I (COI) and 16S rRNA, and the nuclear gene Histone 3 (H3). DNA extraction procedure, PCR amplification options, and sequence obtainment have been previously described in detail in Korshunova et al. (2017a; 2018). Protein-coding sequences were translated into amino acids to verify coding regions and avoid improper base-calling. All new sequences were deposited in GenBank (Suppl. material 1: Table S1, highlighted in bold). Additionally, publicly available sequences of representatives of the genus *Amphorina*, plus data for two *Eubranchus tricolor* (outgroup specimens) were included in the molecular analysis. Sequences were aligned with the MAFFT algorithm (Katoh et al. 2002). Separate analyses were conducted for COI (657 bp), 16S (447 bp), H3 (327 bp), and the concatenated dataset (1431 bp). Evolutionary models for each data set were selected using MrModelTest 2.3 (Nylander et al. 2004). The GTR + I + G model was chosen for the combined full dataset. Two different phylogenetic methods, Bayesian Inference (BI) and Maximum Likelihood (ML), were used to infer evolutionary relationships. Bayesian estimation of posterior probability was performed in MrBayes 3.2 (Ronquist et al. 2012). Four Markov chains were sampled at intervals of 500 generations. Analysis was started with random starting trees and 10^7^ generations. Maximum Likelihood-based phylogeny inference was performed in RAxML 7.2.8 (Stamatakis et al. 2008) with bootstrap in 1000 pseudo-replications. Final phylogenetic tree images were rendered in FigTree 1.4.2 (http://tree.bio.ed.ac.uk). To evaluate the genetic distribution of the different haplotypes a haplotype network was constructed using the Population Analysis with Reticulate Trees (PopART, http://popart.otago.ac.nz) with the TCS network method. The program MEGA7 (Kumar et al. 2016) was used to calculate the uncorrected p-distances. Alignment from the COI of *Amphorina* specimens was processed in Automatic Barcode Gap Discovery (ABGD, available at https://bioinfo.mnhn.fr/abi/public/abgd/abgdweb.html) with the following settings: a prior for the maximum value of intraspecific divergence between 0.001 and 0.1, 10 recursive steps within the primary partitions defined by the first estimated gap, and a gap width of 1.5. COI alignment was analysed separately using both Jukes-Cantor (JC69) and Kimura (K80) proposed models. The distance-based single-locus species delimitation was then used to generate primary species hypotheses, which were tested using the multi-species coalescent-based multi-locus species delimitation, BPP v.3.1. (Yang 2015). In this model, genes evolve inside a species phylogeny, the branches are species, and their properties restrict the gene trees. One of these restrictions is that the divergence times between species have to be more recent than the coalescent times for any genes shared between them, assuming no genetic transfer after speciation (Rannala and Yang 2003). This model can be used for statistical testing of species assignments (Fujita et al. 2012; Rannala 2015) and has been shown to outperform distance methods (Yu et al. 2017). COI, 16S, and H3 were used and the dataset was divided into eight primary species hypotheses to be tested based on the result of the phylogenetic and ABGD analyses, as well as brackish water or oceanic salinity environment, locality and depth of habitat (Suppl. material 1: Table S1). Two analyses (X and Y) with different population size (θs) and divergence time (τ0) priors were preformed, using the same settings and priors as in Martinsson and Erséus (2018) (X: θ 2,400, τ0 2,200; Y: θ 2,1000, τ0 2,200). All analyses were performed three times to confirm consistency between runs. We considered species delimited with a PP ≥ 0.90 in all analyses to be well supported. For clusters with a PP < 0.90, we accepted the best-supported more inclusive species. Bathymetric data were evaluated statistically using nonparametric Mann-Whitney rank sum tests.

**Figure 1.**
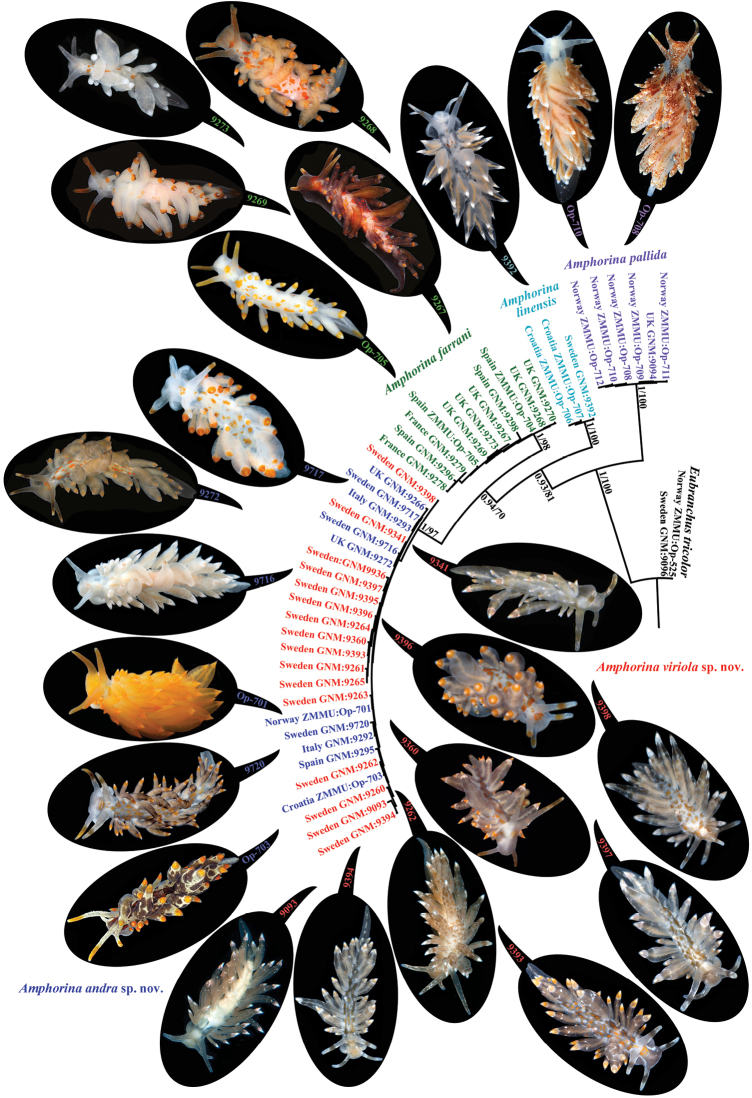
Phylogenetic relationships of *Amphorina* nudibranchs based on the COI+16S+H3 concatenated dataset inferred by Bayesian Inference (BI). The posterior probabilities from BI/ bootstrap values for Maximum Likelihood (ML) are shown.

**Figure 2.**
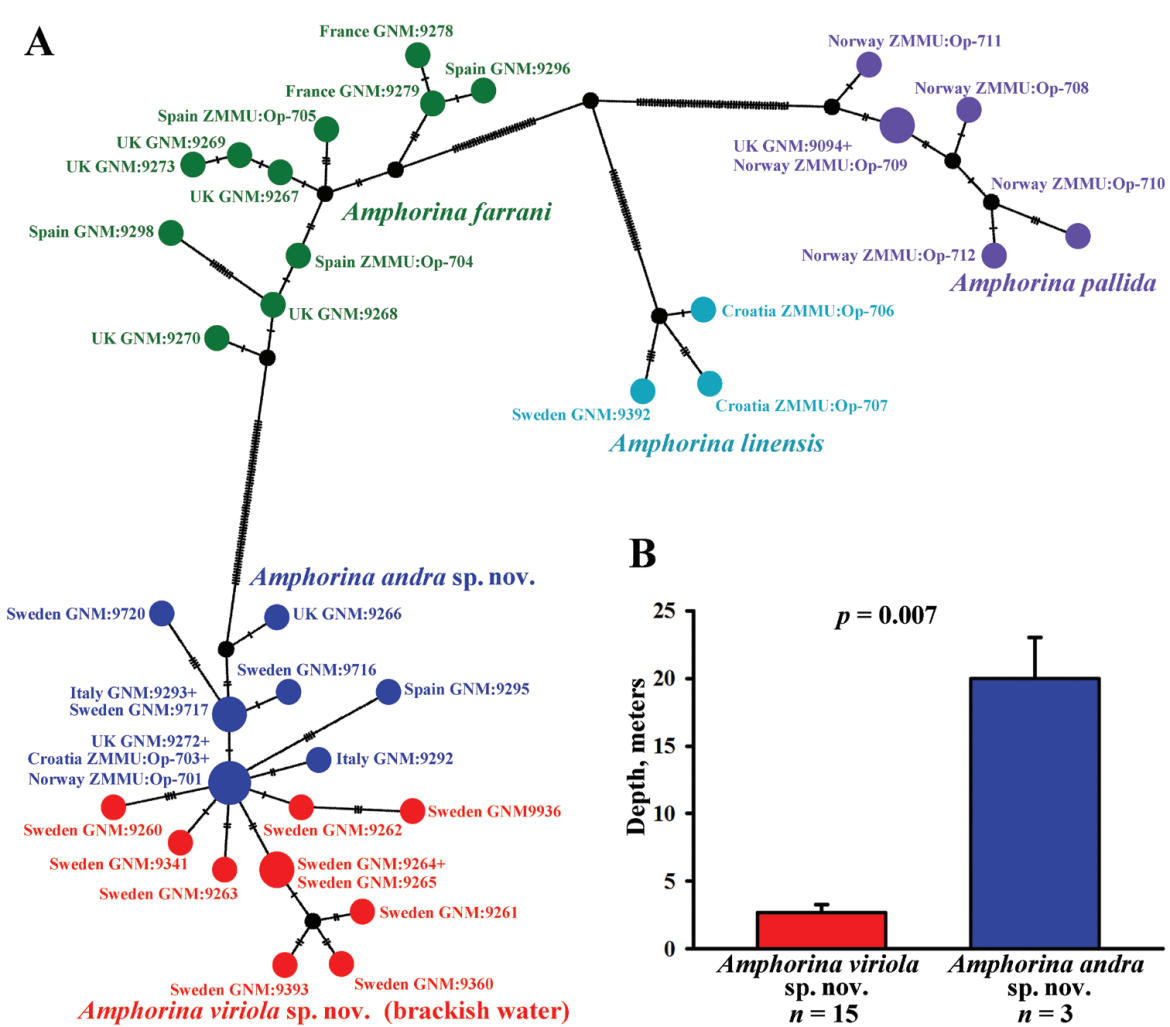
The haplotype network based on cytochrome c oxidase subunit I (COI) molecular data showing genetic mutations occurring within species of the genus *Amphorina* (**A**). Statistical test of the reliability of the bathymetric distribution patterns (and correlated with depths of brackish and marine environments) of *A. viriola* sp. nov. (red bar) and *A. andra* sp. nov. (blue bar) in Swedish waters (**B**). All specimens of *A. viriola* sp. nov. occur strictly in a very shallow brackish water layer above the halocline (salinity usually ca. 24–25‰), whereas in the same geographic region *A. andra* sp. nov. occur only below the halocline (at ca. 15 m depth) in waters with more stable oceanic salinity at 34–35‰.

**Figure 3.**
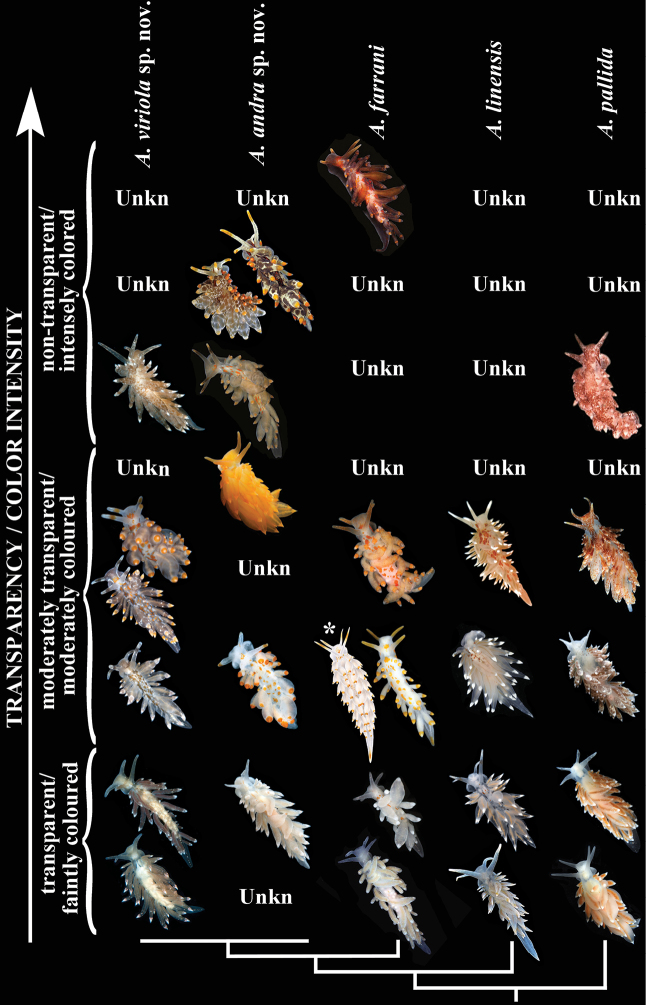
Periodic-like presentation of colour variation patterns among all species of the genus *Amphorina*, represented as vertical rows. Three main periods (horizontal rows), each with several subperiods are presented with spotless body/colourless forms at the bottom to forms with a maximal number of spots/coloured body at the top. Note that different species fundamentally display similar colouration patterns, but not all species display all colourations, so some morphs in particular species (e.g., forms with extensive surface pigmentation and dark body in *A. farrani*, *A. linensis*, and *A. pallida*) can either be eventually discovered or do not exist, by some further constraints of the developmental system. Non-observed forms for each particular species are indicated as “unkn” = “unknown”). * = Image from Alder and Hancock 1845.

**Figure 4.**
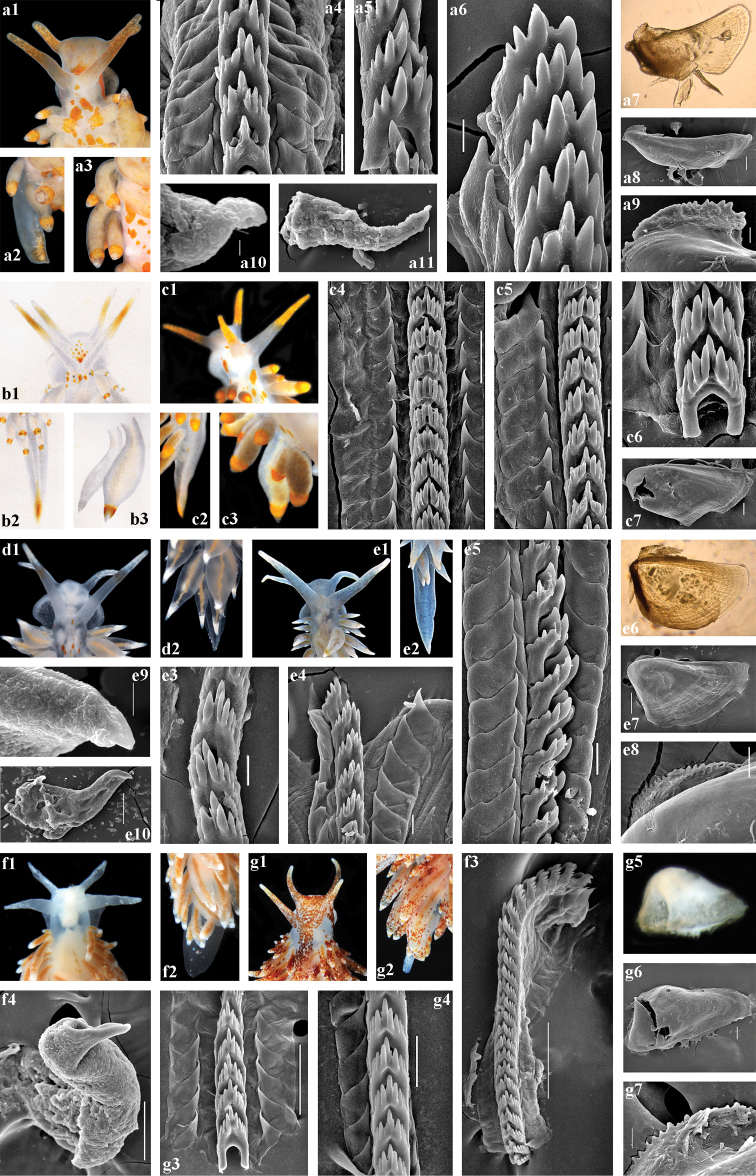
*Amphorina farrani* (Alder & Hancock, 1844) (**a–c**), *A. linensis* (Garcia-Gomez, Cervera & Garcia, 1990) (**d, e**) and *A. pallida* (Alder & Hancock, 1842) (**f, g**). **a***A. farrani*, neotype GNM9268, UK, a1, head; a2, tail; a3, cerata; a4, posterior part of radula (SEM, scale bar 20 μm); a5, posterior part of radula (10 μm); a6, anterior part of radula (10 μm); a7, jaw (light microscopy); a8, jaw (SEM, 100 μm); a9, jaw details (20 μm); a10, details of stylet (3 μm); a11, penis with stylet (30 μm) **b***A. farrani*, image from description of *Eolis farrani* in Alder and Hancock 1845 (not in copyright), b1, head; b2, tail with orange-yellow colouration; b3, cerata **c***A. farrani*, France, Mediterranean, (external data – ZMMU Op-702), с1, head; с2, tail; с3, cerata; (internal data – GNM9278), с4, posterior part of radula (50 μm); с5, anterior part of radula (20 μm); с6, anterior part of radula (20 μm); с7, jaw (100 μm) **d***A. linensis* GNM9392, Sweden, d1, head; d2, tail and cerata **e***A. linensis* ZMMU Op-707, Mediterranean, Croatia, e1, head; e2, tail and cerata; e3, posterior part of radula (20 μm); e4, anterior part of radula (20 μm); e5, anterior part of radula (20 μm); e6, jaw (light microscopy); e7, jaw (SEM, 200 μm); e8, jaw details (50 μm); e9, stylet details (10 μm); e10, penis with stylet (100 μm) **f***A. pallida* ZMMU Op-710, Norway, f1, head; f2, tail and cerata; f3, radula (100 μm); f4, penis with stylet (100 μm) **g***A. pallida* ZMMU Op-712, Norway, g1, head; g2, tail and cerata; g3, posterior part of radula (100 μm); g4, anterior part of radula (30 μm); g5, jaw (light microscopy); g6, jaw (SEM, 100 μm); g7, jaw details (20 μm).

**Figure 5.**
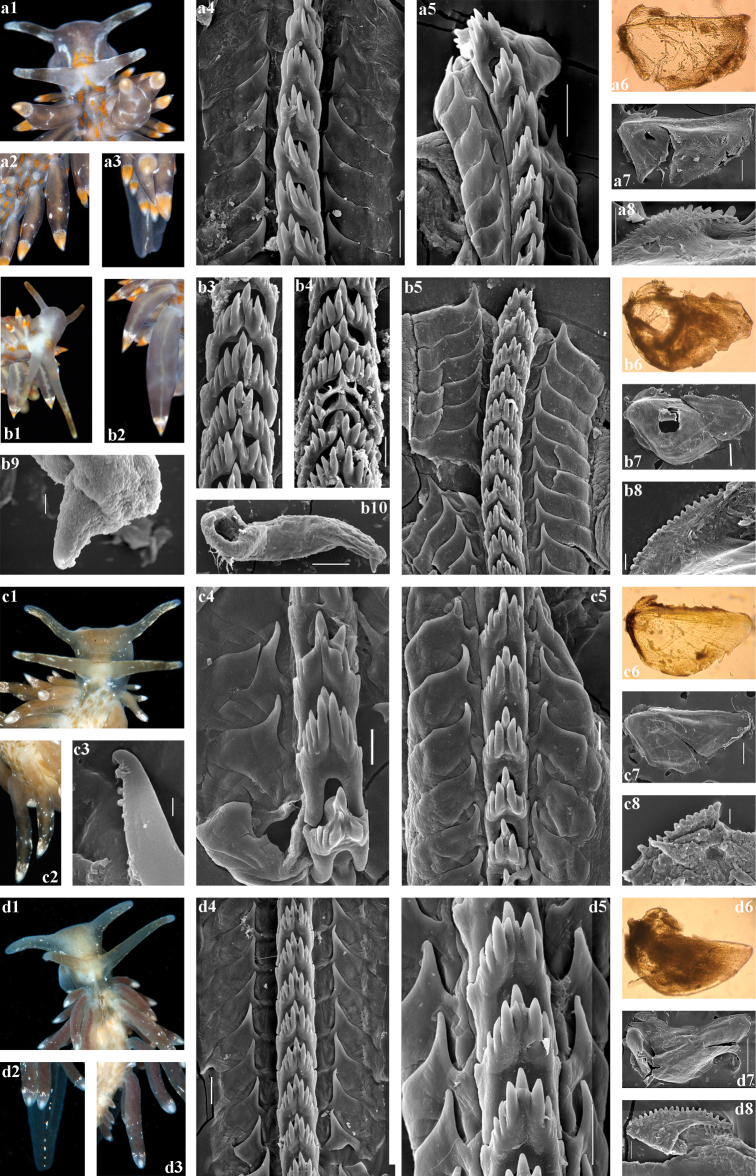
*Amphorina viriola* sp. nov., Sweden. **a***A. viriola* sp. nov., holotype GNM9393, a1, head; a2, cerata; a3, tail; a4, posterior part of radula (30 μm); a5, anterior part of radula (30 μm); a6, jaw (light microscopy); a7, jaw (SEM, 100 μm); a8, jaw details (30 μm) **b***A. viriola* sp. nov., paratype GNM9360, b1, head; b2, cerata; b3, posterior part of radula (10 μm); b4, posterior part of radula (20 μm); b5, anterior part of radula (50 μm); b6, jaw (light microscopy); b7, jaw (200 μm); b8, jaw details (20 μm); b9, stylet details (10 μm); b10, penis with stylet (100 μm) **c***A. viriola* sp. nov., paratype GNM9263, с1, head; с2, cerata; с3, apical part of lateral teeth with possible denticles (1 μm); с4, posterior part of radula (20 μm); с5, anterior part of radula (20 μm); с6, jaw (light microscopy); с7, jaw (SEM, 200 μm); с8, jaw details (20 μm) **d***A. viriola* sp. nov., paratype GNM9260, d1, head; d2, tail; d3, cerata; d4, posterior part of radula (30 μm); d5, anterior part of radula (30 μm); d6, jaw (light microscopy); d7, jaw (300 μm); d8, jaw details (30 μm).

**Figure 6.**
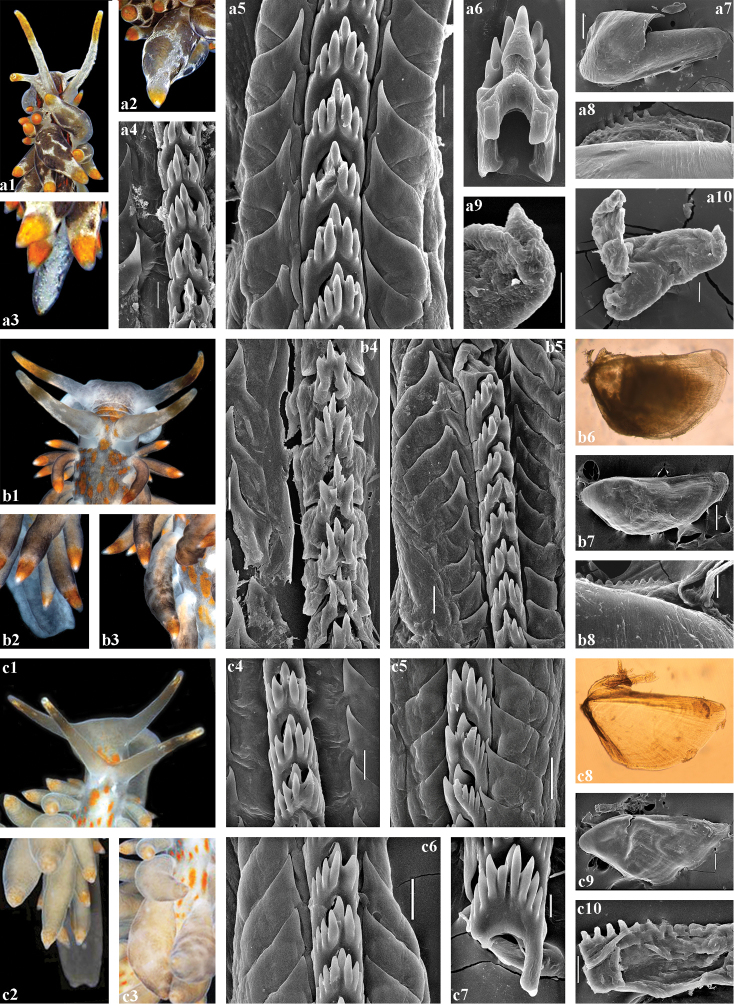
*Amphorina andra* sp. nov. **a***A. andra* sp. nov., paratype ZMMU Op-703, Croatia, a1, head; a2, cerata; a3, tail; a4, posterior part of radula (20 μm); a5, anterior part of radula (20 μm); a6, posterior central tooth (10 μm); a7, jaw (200 μm); a8, jaw details (50 μm); a9, stylet details (30 μm); a10, stylet (30 μm) **b***A. andra* sp. nov., paratype GNM9720, Sweden, b1, head; b2, tail; b3, cerata; b4, posterior part of radula (20 μm); b5, anterior part of radula (20 μm); b6, jaw (light microscopy); b7, jaw (200 μm); b8, jaw details (20 μm) **c***A. andra* sp. nov., paratype GNM9272, UK, с1, head; с2, tail; с3, cerata; с4, posterior part of radula (20 μm); с5, posterior part of radula (10 μm); с6, anterior part of radula (20 μm); с7, anterior part of radula (20 μm); с8, jaw (light microscopy); с9, jaw (SEM, 100 μm); с10, jaw details (20 μm).

**Figure 7.**
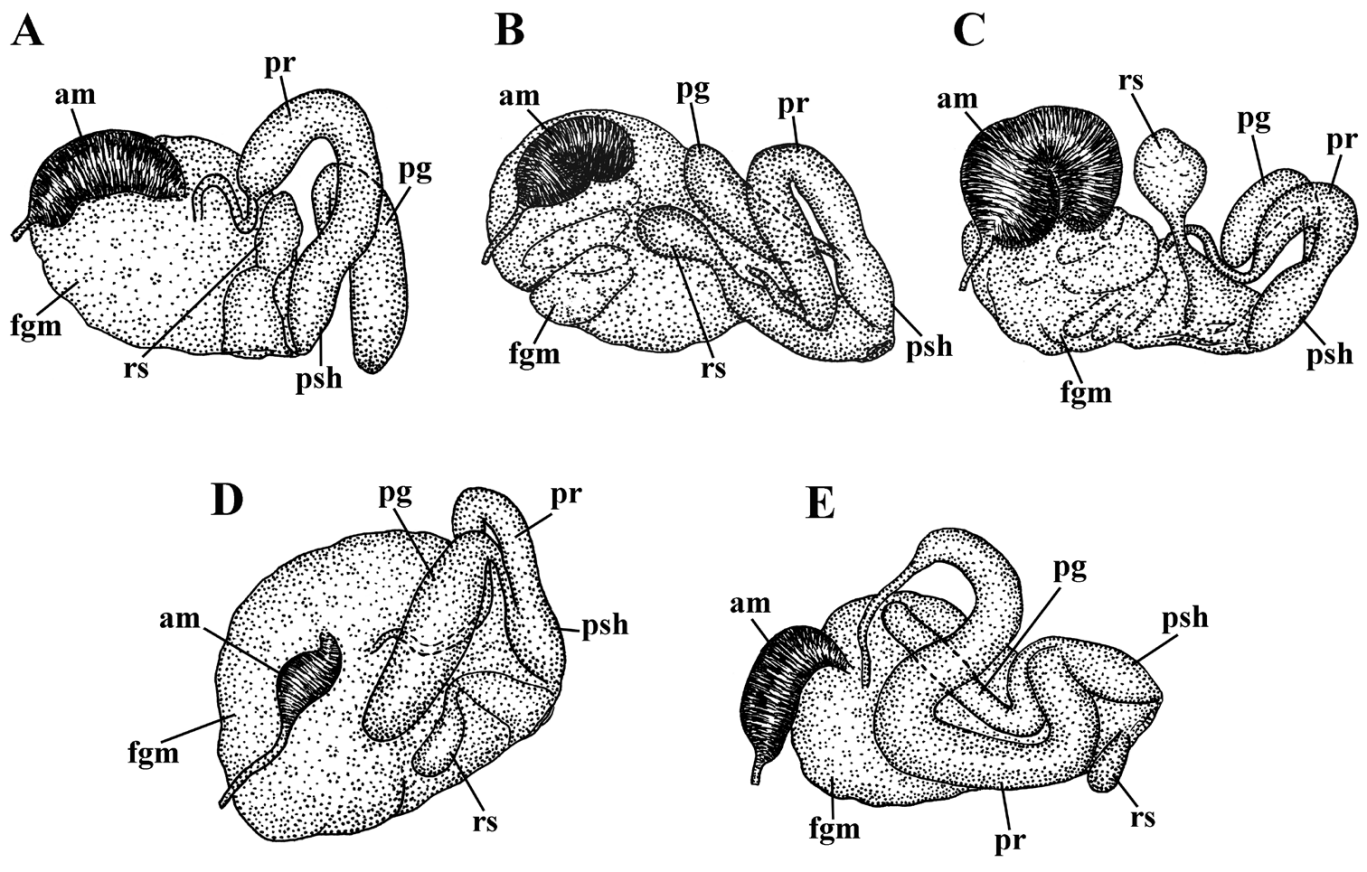
Reproductive systems, schemes. **A***Amphorina farrani***B***Amphorina viriola* sp. nov. **C***Amphorina andra* sp. nov. **D***Amphorina linensis***E***Amphorina pallida*. Abbreviations: am–ampulla, fgm–female gland mass, pg–supplementary (“penial”) gland, pr–prostate, psh–penial sheath, rs–receptaculum seminis.

The molecular phylogenetic and delimitation methods were combined with morphological data (Figs 1–7) to build periodic-like rows when similar colour varieties within each species were aligned using calibration by the degree of light to dark surface pigmentation and transparency of body tissue (Fig. 3), and where these similar forms establish several horizontal rows of similar looking specimens within each species.

## Results

### Molecular analysis

Phylogenetic analysis was performed using 46 specimens of the genus *Amphorina*, and two *Eubranchus tricolor*. Bayesian Inference (BI) and Maximum Likelihood (ML) analyses based on the combined dataset yielded similar results (Fig. 1).

*Amphorina pallida* and *A. linensis* species clustered in separate highly supported clades (PP = 1, BS = 100; Fig. 1). Eleven *A. farrani* specimens from the UK, France, and Spain clustered in a well-supported clade (PP = 1, BS = 98 %) and sister to another well-supported clade (PP = 1, BS = 97) containing the new species of the genus *Amphorina*.

Initially, Automatic Barcode Gap Discovery (ABGD) was used for species delimitation. ABGD analysis of the COI dataset run with two different models for species of the genus *Amphorina* and *Eubranchus tricolor* revealed five potential species: *A. pallida*, *A. linensis*, *A. farrani*, “*A.* sp. nov.”, and *E. tricolor*. Nevertheless, the data of external and internal morphology of specimens in the clade “*Amphorina* sp. nov.” and features of their ecology allowed us to make the assumption that the clade “*Amphorina* sp. nov.” is composed of a complex of species. ABGD analysis underestimated species diversity among species with low divergence and is recommended as a first grouping hypothesis but it is not robust for definitive species delimitation proof (Puillandre et al., 2012; Suárez-Villota et al. 2018).

Analysis of multi-locus genomic sequence data under the multispecies coalescent model was conducted. The sequences were divided into an eight-species scenario (Suppl. material 1: Table S1). In analysis X, the six species model (O, P, L, F, CD, AB) is preferred with a mean PP of 0.93. In analysis Y, the same six species model is also preferred with a higher mean PP of 0.99. Based on the high support for separation, the conclusion is that these two groups represent an “*Amphorina* sp. nov.” clade: *A. viriola* sp. nov. (AB), and *A. andra* sp. nov. (CD). It is important to note that all brackish water specimens from different localities were recognised as single group (AB). Specimens from the same locality (Smögen), but living deeper in oceanic saltwater below the halocline, were recognised as a separate group (CD). In the COI haplotype network (Fig. 2) haplotype groups are shown based on the results of the multilocus species delimitation analysis.

### Integrating morphological and molecular data within a periodic-like framework

The molecularly and morphologically confirmed specimens of all species of the genus *Amphorina* were arranged as follows: by vertical rows indicating the topology of five recognised species according to the phylogenetic tree and by horizontal rows (periods) indicating a reduction of the transparency of the studied specimens of all species due to increasing colouration intensity (Fig. 3). Three main periods are recognised (with several subdivisions): transparent/faintly coloured; moderately transparent/coloured; and non-transparent/intensely coloured. Bottom row (period) – very little to no epidermal pigmentation, the colour is formed due to body colouration (which is almost transparent), and partly by the colouration of the digestive gland and other internal organs. With the succession of the periods upwards, there is an increasing appearance of epidermal pigmentation of several colours: opaque white, yellow, orange, red, and brown. Therefore, the higher the row, the more epidermal pigment coverage there is with a more homogeneously coloured and less transparent body. The penultimate row includes an intensely dark maroon colouration, that so far is known only for *Amphorina farrani*. Unknown, but potentially existing forms, are indicated by “unkn” for every species.

## Systematics

### Phylum Mollusca

**Order Nudibranchia Cuvier, 1817**

**Family Eubranchidae Odhner, 1934**

#### Genus Amphorina

Taxon classificationAnimalia NudibranchiaEubranchidae

Quatrefages, 1844

1C4FD42F-6A15-5B35-BC6E-1ACFF729D3AF

Amphorina Quatrefages 1844: 145–146; Martynov 1998: 774.
Amphorina Non sensu Trinchese 1877–1879 and auctt. (mixed with Trinchesia spp.)

##### Type species.

*Amphorina alberti* Quatrefages, 1844.

##### Diagnosis.

Ceratal rows not branched. Up to six anterior ceratal rows (commonly no more than four). Cerata without tubercles, usually considerably swollen. Rhinophores smooth. Pharynx and jaws moderately broad. Central teeth with central cusp adpressed by adjacent lateral denticles. Prostate thick, readily distinct from vas deferens, moderate in length to very long. Distal receptaculum seminis oval to elongate on a moderately long stalk. Supplementary gland inserts into penis commonly via a narrowing stalk. Penis conical, always with a relatively short, slightly curved, hollow stylet.

##### Species composition.

In this study, we confirm that genus *Amphorina* currently includes the following five species: *A. andra* sp. nov., *A. farrani* (Alder & Hancock, 1844), *A. linensis* (Garcia-Gomez, Cervera & Garcia, 1990), *A. pallida* (Alder & Hancock, 1842), and *A. viriola* sp. nov.

#### Amphorina farrani

Taxon classificationAnimalia NudibranchiaEubranchidae

(Alder & Hancock, 1844)

11AB49F6-4B44-54C4-BEEE-AA30BB4EE074

Figures 1 , 2 , 3 , 4a–c , 7A

Eolis farrani Alder & Hancock, 1844: 164–165; Alder & Hancock, 1845: fam 3, pl. 35.
Galvina farrani (Alder & Hancock, 1844): Bergh 1873: 622; Colgan 1914: 183–185.
Cavolina farrani (Alder & Hancock, 1844): Gray J.E. 1857: 226.
Eubranchus farrani (Alder & Hancock, 1844): O’Donoghue 1926: 128.
Eubranchus farrani : sensu Edmunds & Kress, 1969: forms A & B only: 890, fig. 2A, B.
Amphorina farrani (Alder & Hancock, 1844): Martynov 1998: 775.
Amphorina alberti Quatrefages, 1844: 146–151, pl. 3, fig. 5, pl. 4, fig. 3.
Aeolis adelaidae Thompson, 1860: 49.
Eolis robertianae M’Intosh, 1865: 393.
Eolis tricolor sensu Friele and Hansen 1876, non Forbes 1838.
Amphorina alberti Non sensu Trinchese 1877–1879 and auctt. (= Trinchesia spp.)
Eubranchus farrani Non all forms of Eubranchus farrani sensu Edmunds and Kress 1969 (mixture of several species)
Eubranchus farrani Non sensu Schmekel and Portmann 1982: 241–243, taf. 14, figs 1–3, abb. 7.78 (= Amphorina andra sp. nov. + mixture of species).

##### Material examined.

***Neotype***. NE Atlantic, the United Kingdom, Cornwall, Newlyn Marina, (50°06'10.00"N, 05°32'45.00"W), 10–20 m depth, stones with hydroids, 12 Aug 2015, coll. David Fenwick (GNM Gastropoda – 9268, preserved length 4.5 mm).

***Other specimens***. NE Atlantic, the United Kingdom, Cornwall, Newlyn Marina (50°06'10.00"N, 05°32'45.00"W), 10–20 m depth, stones with hydroids, 12 Aug 2015, coll. David Fenwick (GNM Gastropoda – 9267, preserved length 3 mm, GNM Gastropoda – 9269, preserved length 2.5 mm, GNM Gastropoda – 9270, preserved length 4 mm, GNM Gastropoda – 9271, preserved length 3.5 mm), GNM Gastropoda – 9273, preserved length 5.5 mm). Mediterranean Sea, France, Banyuls (42°28'58.00"N, 03°08'13.00"E), 10–12 m depth, 07 Sept 2010, coll. Alexander Martynov and Tatiana Korshunova, one specimen (ZMMM Op-702, 9.5 mm in length, live, preserved length 4 mm). NE Atlantic, Spain, Vigo (42°24'06.00"N, 08°72'07.00"E), 5–10 m depth, 04 Sept 2010, coll. Tatiana Korshunova and Alexander Martynov, one specimen (ZMMU Op-704, 7.5 mm in length, live, preserved length ca. 4 mm). NE Atlantic, Spain, Vigo (42°24'06.00"N, 08°72'07.00"E), 5–10 m depth, 04 Sept 2010, coll. Tatiana Korshunova and Alexander Martynov, one specimen (ZMMU Op-705, 6.5 mm in length, live, preserved length 3 mm).

##### Diagnosis.

Body up to 20 mm; large dorsal pigment spots, if present, yellow-orange, bright; in specimens with bright yellow-orange spots on dorsal side and cerata, a distinct yellow-orange spot or stripe on the tail is always present; completely pale specimens lacking tail spot or stripe; no light pinkish subapical ring on cerata; absence of punctuated white line on edge of foot; cerata commonly moderate in width without distinctly attenuated apices; digestive gland in cerata relatively broad without distinct short branches; up to four anterior rows of cerata; radular formula 35–38 × 1.1.1, copulative stylet short and slightly bent at the top, receptaculum seminis pear-shaped without short distinct stalk between reservoir and short wide base.

##### Description.

***External morphology.*** The live length of the neotype is ca. 10 mm (Fig. 1, GNM: 9268, Fig. 4a). The length of adult specimens may reach 20 mm and more. The body is narrow. The rhinophores are smooth and 1.5–2 times longer than the oral tentacles. The cerata are relatively long, swollen. Ceratal formula of the neotype: right (1, 3, 3; anus, 3, 3, 2, 1) left (1, 3, 4; anus, 4, 3, 2, 1). The foot is narrow, anteriorly without foot corners.

***Colour.*** There are three main colour morphs with several subdivisions of colour variations (Fig. 3), from a completely pale body and cerata with reduced orange-yellow pigment spots, to specimens with distinct orange-yellow spots on the body and a broad subapical orange-yellow ring on each ceras, sometimes with a deep maroon body colour. No specimens with blotches of blackish surface pigmentation have been observed, nor any specimens with uniformly bright orange body. Specimens with distinct orange-yellow spots on the body always have an orange-yellow spot or stripe on the tip of the tail. The upper part of the rhinophores is covered with orange pigment and scattered small white dots without a light pinkish pigment ring. The oral tentacles are similarly coloured.

***Anatomy.*** Digestive system (Fig. 4, a4–a11, c4–c7). The jaws are triangularly ovoid. The masticatory processes of the jaws bear a single row of ca. 20–25 distinct denticles. The radular formula in three studied specimens is 35–38 × 1.1.1. The radular teeth are yellowish. The central tooth is narrow, with a low cusp and 3–7 lateral denticles, including smaller intercalated denticles that may occur in different parts of the tooth.

***Reproductive system.*** (Fig. 7A). The ampulla is moderate in length and swollen (Fig. 7A, am). The prostate is distinct, moderately long and wide (Fig. 7A, pr). The prostate transits to a penial sheath, which contains a conical penis with a short, chitinous, very slightly curved stylet (Fig. 4, a10, a11). A supplementary (“penial”) gland is relatively short and inserts into the base of the penis (Fig. 7A, pg). The receptaculum seminis is relatively small, irregularly oval, which transits directly to a large widened base without a distinct stalk (Fig. 7A, rs). The female gland mass includes mucous and capsular glands (Fig. 7A, fgm).

##### Distribution and habitats.

Mediterranean Sea and all European Atlantic coasts to Norway, from very shallow water (0–0.5 m) to ca. 25 m. On the Swedish west coast, it lives below the halocline (15–25 m).

##### Remarks.

Morphologically *A. farrani* differs from the closely related *A. andra* sp. nov. (which also inhabits waters with normal oceanic salinity) and the brackish *A. viriola* sp. nov. by the presence of orange-yellow colouration on the tail in spotted forms (see Discussion), the absence of forms with blackish surface pigmentation, and uniformly bright orange forms (Fig. 3). From the exclusively brackish-water species *A. viriola* sp. nov., *A. farrani* additionally differs by the absence of light pinkish subapical ceratal colouration. From *A. linensis*, *A. farrani* differs by the absence of a distinct dotted white line along the foot edge, orange-yellow and not reddish orange spots (in spotted forms), fewer ceratal rows and the shape of the cerata. From *A. pallida*, *A. farrani* differs by the larger size of dorsal spots (in spotted forms), the absence of small orange-brownish or brown spots on the cerata, and the smaller number of anterior ceratal rows. In *A. farrani*, the largest possible number of lateral denticles so far detected on the central teeth is up to seven, compared to up to five in *A. andra* sp. nov. and up to six in *A. viriola* sp. nov. The reproductive system of *A. farrani* differs from all *Amphorina* species (including *A. andra* sp. nov.) by the presence of an oval receptaculum seminis with a broad base but without a distinct stalk; from *A. viriola* sp. nov. and *A. linensis* by the shape of ampulla; from *A. pallida* by a considerably shorter prostate gland.

The species *Amphorina alberti* Quatrefages, 1844 was described the same year as *A. farrani* (Alder & Hancock, 1844) and morphologically they are essentially similar. Unfortunately, although the name *A. alberti* was referenced in some publications as a eubranchid (e.g., Bergh 1877; Iredale and O’Donoghue 1923) it was also incorrectly applied to several non-eubranchid *Trinchesia* species (Trinchese 1877–1879; Bergh 1882). *Eolis farrani* was treated as a eubranchid, although its synonymy was partially cleared up only after the mid-20^th^ century (e.g., Edmunds and Kress 1969; Thompson and Brown 1984; compared with the incorrect lumping synonymy of *A. farrani* in Iredale and O’Donoghue 1923). Therefore, in order to avoid confusion, the name *A. alberti* Quatrefages, 1844 was previously suppressed under plenary powers in favour of precedence of the name *Eolis farrani* Alder & Hancock, 1844 (ICZN 1966). At the same time, the genus name *Amphorina*, per se, in the original sense of Quatrefages (1844) was left as a potentially available genus name for the group of “*Eubranchus farrani*” (Heppel 1964), and that previous proposal corroborates well with the modern integrative data presented in this study.

Minimum uncorrected p-distances of the COI marker which separate *A. farrani* from *A. viriola* sp. nov., *A. andra* sp. nov., *A. linensis*, and *A. pallida* are 8.92%, 9.59%, 10.05%, and 14.31% respectively.

#### Amphorina viriola sp. nov.

Taxon classificationAnimalia NudibranchiaEubranchidae

860DEC1E-AB9B-5995-AD2D-5B84C476B584

http://zoobank.org/D56F2608-384E-4C02-9BD0-8B66A679A4A9

Figures 1 , 2 , 3 , 5 , 7B

##### Material examined.

***Holotype.*** NE Atlantic, Skagerrak, Sweden, Region Västra Götaland, Bohuslän county, Ide fjord, close to Svarte Jan lighthouse (59°06'30"N, 11°19'30"E), 4–6 m depth, 21 Dec 2016, coll. Klas Malmberg (GNM Gastropoda – 9393, 6 mm in length, live, preserved length 3 mm). ***Paratypes*.** NE Atlantic, Skagerrak, Sweden, Region Västra Götaland, Bohuslän county, town of Lysekil, public marina, Dock D (58°16'00"N, 11°26'00"E), 0.1–0.5 m depth, a mix of algae on floating blocks, 09 May 2015, coll. Klas Malmberg, seven specimens (GNM Gastropoda – 9093, 6 mm in length, live, GNM Gastropoda – 9260, preserved length 5.5 mm, GNM Gastropoda –9261, 7 mm in length, live, preserved length 6.5 mm, GNM Gastropoda –9262, 8 mm in length, live, preserved length 6.5 mm, GNM Gastropoda –9263, 8 mm in length, live, preserved length 5 mm, GNM Gastropoda –9264, 6 mm in length, live, preserved length 5 mm, GNM Gastropoda –9265, 7 mm in length, live, preserved length 5.5 mm). NE Atlantic, Skagerrak, Sweden, Region Västra Götaland, Bohuslän county, town of Smögen, Kleven, Smögen Dyk och Upplevelse Dive centre (58°21'08.8"N, 11°13'40.6"E), 3 m depth, 25 Mar 2017, one specimen (GNM Gastropoda –9341, 7 mm in length, live). NE Atlantic, Skagerrak, Sweden, Region Västra Götaland, Bohuslän county, town of Smögen, Kleven, Smögen Dyk och Upplevelse Dive centre (58°21'30.8"N, 11°13'31.0"E), 4–4.5 m depth, 01 Apr 2017, coll. Sebastian Spora, one specimen (GNM Gastropoda –9360, 7 mm in length, live, preserved length 5 mm). NE Atlantic, Skagerrak, Sweden, Region Västra Götaland, Bohuslän county, Ide fjord, close to Svarte Jan lighthouse, five specimens (59°06'30"N, 11°19'30"E), 4–6 m depth, 21 Dec 2016, coll. Klas Malmberg (GNM Gastropoda – 9394, 8 mm in length, live, preserved length 4 mm, GNM Gastropoda – 9395, 7 mm in length, live, preserved length 3 mm, GNM Gastropoda – 9396, 6 mm in length, live, preserved length 3.2 mm, GNM Gastropoda – 9397, 8 mm in length, live, preserved length 2 mm, GNM Gastropoda – 9398, 8 mm in length, live, preserved length 7 mm). NE Atlantic, Skagerrak, Sweden, Region Västra Götaland, Bohuslän county, Ide fjord, close to Svarte Jan lighthouse (59°06'30"N, 11°19'30"E), depth unknown, 2018–2019, coll. Mats Larsson, Michael Lundin (GNM Gastropoda – 9936).

##### Diagnosis.

Body up to ca. 12 mm; large dorsal pigment spots, if present, yellow-orange, dull; in specimens with yellow-orange spots on body and cerata there is never any yellow-orange pigment spot or stripe on the tail, but there might be a median whitish line or broken line on the tail; completely pale specimens lack tail spot; light pinkish subapical ring on cerata present; absence of white punctuated line on external edge of foot; cerata commonly moderate in width without distinctly attenuated apices; digestive gland in cerata relatively broad without distinct short branches; up to four anterior rows of cerata; radular formula 31–47 × 1.1.1, copulative stylet relatively long and almost straight, at the top, receptaculum seminis pear-shaped with short distinct stalk between reservoir and long base.

##### Etymology.

*viriola*, Lat. small bracelet, referring to the light pinkish subapical pigment ring on the cerata.

##### Description.

***External morphology*.** The live length of the holotype is 6 mm (Fig. 1, GNM: 9393; Fig. 5a). The length of adult specimens may reach 10–12 mm. The body is narrow. The rhinophores are smooth and 1.5–2 times longer than the oral tentacles. The cerata are relatively long, swollen. Ceratal formula of the neotype: right (2, 4, 4; anus, 3, 3, 2, 2, 1) left (2, 3, 3; anus, 3, 2, 2, 1, 1). The foot is narrow, anteriorly without foot corners.

***Colour*.** There are three main colour morphs with several subdivisions of colour variations (Fig. 3), from a completely pale body and cerata without orange-yellow pigment spots on the body, to specimens with dull brownish orange-yellow spots. In the specimens with such spots there is never any orange-yellow colouration on the tail, but there can be a median whitish line or broken line on the tail. Specimens with greyish surface pigmentation are sometimes found, but not with blackish, non-transparent pigmentation (Fig. 3). No specimens with uniformly orange colour have been observed. The tips of the cerata may have orange-yellow pigmentation or lack pigment, leaving the cnidosacs visible. A light pinkish subapical ring on the cerata is usually present, or at least noticeable by some pinkish pigment dots. Absence of a punctuated white line on the edge of the foot. The upper part of the rhinophores are commonly covered with brownish to dark orange pigment and dispersed small white dots. The oral tentacles are similarly coloured.

***Anatomy*.** Digestive system (Fig. 5 a4–a8, b3–b8, c3–c8, d4–d8). The jaws are triangularly ovoid. The masticatory processes of the jaws bear a single row of ca. 15–21 distinct denticles. The radular formula in four studied specimens is 31–47 × 1.1.1. The radular teeth are yellowish. The central tooth is narrow, with a low cusp and 4–6 lateral denticles, including smaller intercalated denticles that may occur in different parts of the tooth.

***Reproductive system*.** (Fig. 7B). The ampulla is moderate in length and swollen (Fig. 7B, am). The prostate is distinct, moderately long and wide (Fig. 7B, pr). The prostate transits to a penial sheath, which contains a conical penis with a short, chitinous, almost straight stylet (Fig. 5, b9, b10). A supplementary (“penial”) gland is relatively short and inserts into the base of the penis (Fig. 7B, pg). The receptaculum seminis is moderate, pear-shaped (Fig. 7B, rs) with a distinct stalk, which transits to a long broad base. The female gland mass includes mucous and capsular glands (Fig. 7B, fgm).

##### Distribution and habitats.

Swedish northwest Skagerrak coast, in the south from the town of Lysekil at the Gullmar fjord, onwards to Smögen and the Väderö Island archipelago, to the Ide fjord in the north by the border with Norway. It is always found very shallow and above the halocline (situated at 6–7 m depth within the fjords and 15 m outside the fjords), most often from 0.1 to 6 metres depth, commonly on wharf pontoons in the marina. Inhabits exclusively the brackish water layer, salinity-range: ordinarily ca. 24–25‰ but may vary from 12 to 30‰.

##### Remarks.

Morphologically the brackish water-living *A. viriola* sp. nov. differs from the closely related *A. andra* sp. nov. by the presence of light pinkish subapical rings on the cerata, the absence of forms with non-transparent blackish pigmentation, or any forms with uniform orange colour (Fig. 3), a larger range of the number of lateral denticles on central radular teeth, a considerably smaller ampulla and an elongated and pear-shaped receptaculum seminis. From *A. farrani*, *A. viriola* sp. nov. differs by the absence of a yellow or orange median stripe on its tail, and the absence of forms with black surface pigmentation (Fig. 3). From *A. linensis*, *A. viriola* sp. nov. differs by the absence of a distinct white line (sometimes dotted) along the foot edge, orange-yellow and not reddish orange spots (in spotted forms), a smaller number of ceratal rows and the shape of the cerata, the shape and size of the ampulla and receptaculum seminis. From *A. pallida*, *A. viriola* sp. nov. differs by the larger size of dorsal spots (in spotted forms), fewer anterior ceratal rows, and the shape and size of the ampulla and receptaculum seminis.

Minimum uncorrected p-distances of the COI marker which separate *A. viriola* sp. nov. from *A. farrani*, *A. andra* sp. nov., *A. linensis*, and *A. pallida* are 8.92%, 0.15%, 9.15%, and 14.08% respectively.

#### Amphorina andra sp. nov.

Taxon classificationAnimalia NudibranchiaEubranchidae

07F8A48A-1048-564E-AC1A-FC398D318DF4

http://zoobank.org/91211302-2EFC-4BC5-913E-1DE47D8DE2FA

Figures 1 , 2 , 3 , 6 , 7C

Eubranchus farrani : sensu Schmekel and Portmann 1982: 241–243, taf. 14, Figs 1–3, abb. 7.78 (= Amphorina andra sp. nov. + mixture of species).
Eubranchus farrani : sensu Trainito and Doneddu 2014: 117 (four lower figs), non Alder & Hancock, 1844.
Eubranchus farrani : sensu Prkić et al. 2018: 347, fig. 3a, b; p. 348, fig. 1a–e; p. 349, figs.1a–d; 350, fig. 1a–d; 351, fig. 1a–d, non Alder & Hancock, 1844.

##### Material examined.

***Holotype***. NE Atlantic, Skagerrak, Sweden, Region Västra Götaland, Bohuslän county, town of Smögen, outermost skerries (58°22'00"N, 11°11'00"E), 15–20 m depth, 29 Apr 2018, coll. Klas Malmberg (GNM Gastropoda – 9717, ca. 12 mm in length, live, preserved length ca. 5 mm).

***Paratypes***. NE Atlantic, the United Kingdom, Scotland, Loch Fyne (55°57'00"N, 05°23'00"W), 5–20 m depth, 24 May 2015, coll. Jim Anderson, one specimen (GNM Gastropoda –9266, preserved length 4 mm). NE Atlantic, the United Kingdom, Cornwall, Newlyn Marina (50°06'10"N, 05°32'45"W), 0–5 m depth, 12 Aug 2015, coll. David Fenwick, one specimen (GNM Gastropoda –9272, preserved length 3.5 mm). Mediterranean, Italy, Lecce (40°25'00"N, 18°16'00"E), 10–20 m depth, 20 Feb 2015, coll. Fabio Vitale, one specimen (GNM Gastropoda –9292, preserved length 2 mm). Mediterranean, Italy, Lecce (40°25'00"N, 18°16'00"E), 10–20 m depth, 05 Aug 2016, coll. Fabio Vitale, one specimen (GNM Gastropoda –9293, preserved length 2 mm). NE Atlantic, Skagerrak, Sweden, Region Västra Götaland, Bohuslän county, town of Smögen, outermost skerries (58°22'00"N, 11°11'00"E), 26 m depth, 29 Apr 2018, coll. Klas Malmberg (GNM Gastropoda – 9716, 12 mm in length, live, preserved length 10 mm). NE Atlantic, Skagerrak, Sweden, Region Västra Götaland, Bohuslän county, town of Smögen, Kleven, Smögen Dyk och Upplevelse Dive centre (58°16'00"N, 11°26'00"E), 15–20 m depth, 29 Apr 2018, coll. Klas Malmberg (GNM Gastropoda – 9720, 11 mm in length, live, preserved length 9 mm). Mediterranean Sea, Croatia, Split, Kašuni (43°50'55"N, 16°37'44"E), 20 m depth, 28 Jan 2018, coll. J. Prkić and Marko Lete, one specimen (ZMMU Op-703, ca. 11 mm in length, live, preserved length 6 mm).

##### Diagnosis.

Body up to at least 20 mm; large dorsal pigment spots, if present, bright yellow-orange or reddish orange; in specimens with yellow-orange or reddish spots on dorsal side and cerata, there is never any yellow-orange spot or stripe on the tail, but there could be a whitish median line on the tail; completely pale specimens lack tail stripe or spot; light pinkish subapical ring on cerata absent; absence of a punctuated white line or row of dots on the edge of foot; cerata commonly moderate in width without distinctly attenuated apices; digestive gland in cerata relatively broad without distinct short branches; up to four anterior rows of cerata; radular formula 30–37 × 1.1.1, copulative stylet very short and conical, receptaculum seminis subcircular with long distinct stalk between reservoir and rapidly widening base.

##### Etymology.

*andra* from Swedish meaning other referring to the separation from *A. viriola*.

##### Description.

***External morphology*.** The live length of holotype is ca. 12 mm (Fig. 1, GNM:9717). The length of adult specimens may reach 20 mm. The body is narrow. The rhinophores are smooth and 1.5–2 times longer than the oral tentacles. The cerata are relatively long and swollen. Ceratal formula of the holotype: right (2; 3; 3; anus, 2, 3, 2, 2) left (2, 3, 3; anus, 3, 2, 2, 1). The foot is narrow, anteriorly without foot corners.

***Colour*.** There are three main and several subdivisions of colour variations (Fig. 3), from a completely pale body and cerata without orange-yellow pigment spots to specimens with dull orange-yellow spots on the body. In specimens with distinct dorsal spots, no distinct orange-yellow colouration on the tail has yet been observed, but there could be a whitish median line on the tail. The dorsal side of the body can be partially to almost completely covered with brown-greyish, dark brown or blackish pigment spots or blotches on some specimens and similar colours can also be present on the cerata. Yet other specimens can be without any blackish surface pigmentation but with a uniformly homogeneous bright orange to golden yellow body colour (Fig. 3). The tips of the cerata can be covered with orange-yellow pigment, or lack pigmentation, in which case the cnidosacs are visible. There is never any light pinkish subapical ring, nor any small subapical pinkish dots on the cerata. There is no distinct punctuated white line on the edge of the foot. The upper part of the rhinophores is commonly covered with orange to yellowish brownish pigment and dispersed small white spots, without the formation of a pinkish pigment ring, occasionally the entire surface of the rhinophores is covered with yellowish orange or brownish pigment. The oral tentacles are similarly coloured.

***Anatomy*.** Digestive system (Fig. 6 a4–a8, b4–b8, c4–c10). The jaws are triangularly ovoid. The masticatory processes of the jaws bear a single row of ca. 19–28 distinct denticles. The radular formula in four studied specimens is 30–37 × 1.1.1. The radular teeth are yellowish. The central tooth is narrow, with a low cusp and 3–5 lateral denticles, including smaller intercalated denticles that may occur in different parts of the tooth.

***Reproductive system*.** (Fig. 7C). The ampulla is large and conspicuously swollen (Fig. 7C, am). The prostate is distinct, relatively short and wide (Fig. 7C, pr). The prostate transits to a penial sheath, which contains a conical penis with a chitinous, very short, broadly conical stylet (Fig. 6, a9, a10). A supplementary (“penial”) gland is relatively short and inserts into the base of the penis (Fig. 7C, pg). The receptaculum seminis is large, subcircular (Fig. 7C, rs) with a distinct long stalk which transits to a large, widened base. The female gland mass includes mucous and capsular glands (Fig. 7C, fgm).

##### Distribution and habitats.

Mediterranean Sea and all European Atlantic coasts to Gulen at the mouth of Hardanger fjord, Norway, also possibly further north to the Trondheim fjord (Klas Malmberg, personal observation). Salinity-range: 33 to 35‰, ordinary oceanic salinity, or close to it. On the Swedish west coast, it lives below the halocline. In areas without a halocline and in more oceanic environments, it can be found closer to the surface or intertidally. In Croatia it is quite common from very shallow water (0–0.5 m) to ca. 20 m.

##### Remarks.

Morphologically this inhabitant of waters with normal to nearly normal ocean salinity, *A. andra* sp. nov., differs from the closely related strict inhabitant of brackish waters, *A. viriola* sp. nov., by the absence of light pinkish subapical rings on the cerata, the presence of forms with blackish surface pigmentation or uniform orange colouration (Fig. 3), a lower range of the number of lateral denticles on the central radular teeth, and a considerably larger, strongly swollen ampulla and subcircular instead of pear-shaped receptaculum seminis. From *A. farrani*, *A. andra* sp. nov. differs by the absence of orange-yellow colouration in spotted forms (see Discussion), and the presence of forms with blackish surface pigmentation on the body and cerata (Fig. 3), *A. andra* sp. nov. differs from *A. linensis* by the absence of a distinct dotted white line along edge of the foot, fewer ceratal rows, the shape of the cerata, and the shape and size of the ampulla and receptaculum seminis. From *A. pallida*, *A. andra* sp. nov. differs by the larger size of the dorsal spots (in spotted forms), fewer anterior ceratal rows, and the shape and size of the ampulla and receptaculum seminis.

Minimum uncorrected p-distances of the COI marker which separate *A. andra* sp. nov. from *A. farrani*, *A. viriola* sp. nov., *A. linensis*, and *A. pallida* are 9.59%, 0.15%, 11.42%, and 14.92% respectively.

#### Amphorina linensis

Taxon classificationAnimalia NudibranchiaEubranchidae

(Garcia-Gomez, Cervera & Garcia, 1990)

E075FF4A-6730-5246-9490-51546DE37E3E

Figures 1 , 2 , 3 , 4d–e , 7D

Eubranchus linensis Garcia-Gomez, Cervera & Garcia, 1990: 585–593.
Amphorina linensis (Garcia-Gomez, Cervera & Garcia, 1990): Martynov 1998: 775.
Eubranchus tricolor : sensu Trainito and Doneddu 2014: 118, non Forbes, 1838.
Eubranchus sp. 1: Prkić et al. 2018: 353–357.

##### Material examined.

NE Atlantic, Skagerrak, Sweden, Västra Götalands län, Bohuslän, Väderöarna Islands (58°33'00"N, 11°02'30"E), 19 m depth, 09 Apr 2017, coll. Klas Malmberg, one specimen (GNM Gastropoda – 9392, 10 mm in length, live, preserved length 4.2 mm). Mediterranean Sea, Croatia, Iž Island, Svežina (44°03'55"N, 15°07'15"E), 5 m depth, 13 Jan 2018, coll. A. Petani and Đani Iglić, two specimens (ZMMU Op-706, preserved length 6.5 mm, ZMMU Op-707, preserved length 6 mm).

##### Diagnosis.

Body up to 30 mm; dorsal spots, if present, reddish orange; in specimens with dorsal and ceratal spots distinct colouration of tail absent; completely pale specimens lack tail stripe or spot; light pinkish subapical ring on cerata absent; presence of distinct line of white pigment, sometimes punctuated, on the edge of the foot; cerata commonly broad with distinctly attenuated apices; digestive gland in cerata relatively thin without distinct short branches; up to six anterior rows of cerata; radular formula 38–61 × 1.1.1, copulative stylet relatively long, slightly bent at the middle, receptaculum seminis elongate oval with moderate distinct stalk between reservoir and rapidly widening base.

##### Description.

***External morphology*.** The length of adult specimens may reach 30 mm. The body is narrow. The rhinophores are smooth and 1.5–2 times longer than the oral tentacles. The cerata are relatively long, very broad, with distinctly attenuated apices. Ceratal formula of the specimen from Sweden (GNM 9392): right (2, 3, 3, 4; anus, 3, 2, 2, 1) left (2, 3, 4; anus, 3, 2, 2, 1). The foot is narrow, anteriorly without foot corners.

***Colour*.** There are three main and eight subdivisions of colour variations (Fig. 3), from a completely pale body and cerata without pigment spots to specimens with very distinct reddish orange pigment spots on the body. In specimens with distinct dorsal spots, there is never any pigmentation on the tail. A distinct white line is present on the external edge of the foot, although this could be broken or punctuated. No specimens found with blackish non-transparent pigmentation on the body, nor any homogeneously orange specimens. Absence of light pinkish subapical ring on cerata. Small white pigment dots of various density can be present on the cerata. The upper part of rhinophores commonly covered with white, relatively dense dots with small insertions of yellowish brownish pigment in some specimens, without the formation of ring-shaped colouration. The oral tentacles are similarly coloured.

***Anatomy*.** Digestive system (Fig. 4, e3–e8). The jaws are triangularly ovoid. The masticatory processes of the jaws bear a single row of ca. 25 distinct denticles. The radular formula in the specimen studied from Croatia (Op-707) is 38 × 1.1.1. The radular teeth are yellowish. The central tooth is narrow, with a low cusp and four or five lateral denticles (three or less on the anteriormost eroded teeth), including smaller intercalated denticles that may occur in different parts of the tooth.

***Reproductive system*.** (Fig. 7D). The ampulla is relatively small, not distinctly swollen (Fig. 7D, am). The prostate is distinct, relatively short and narrow (Fig. 7D, pr). The prostate transits to a penial sheath, which contains a conical penis with a chitinous, very short, broadly conical stylet (Fig. 4, e9, e10). A supplementary (“penial”) gland is relatively long and inserts into the base of the penis (Fig. 7D, pg). The receptaculum seminis is an elongate oval (Fig. 7D, rs) with a moderate distinct stalk between the reservoir and the rapidly widening base. The female gland mass includes mucous and capsular glands (Fig. 7D, fgm).

##### Distribution and habitats.

Mediterranean Sea and all European Atlantic coasts to Sweden and Southwest Norway. On the Swedish west coast, it lives below the halocline.

##### Remarks.

Morphologically *A. linensis* differs from *A. farrani*, *A. viriola* sp. nov., *A. andra* sp. nov., and *A. pallida* by having reddish orange and not orange-yellow pigment spots (in spotted forms), the presence of a distinct, sometimes dotted white line along the foot edge, the shape of the cerata with attenuated apices, and a small ampulla. The present materials are well consistent with the original description of *A. linensis* (Garcia-Gomez et al. 1990) in such key characters as the shape of the cerata, the presence of a distinct white dotted line along the foot and reddish spots in some specimens, and the shape of the receptaculum seminis and prostate, but there are some differences in the number of rows of the radula, most likely due to specimen size differences. Adriatic specimens differ from the Atlantic ones in having a larger size and different colouration. There is often a light blue pigmentation that covers the cerata, rhinophores and oral tentacles, partially or completely, often the whole animal has a bluish appearance. No specimens with dorsal reddish spots have been found so far on the Adriatic coast. Mediterranean specimens of *A. linensis* have been frequently misidentified in the literature as *Eubranchus tricolor*.

Minimum uncorrected p-distances of the COI marker which separate *A. linensis* from *A. viriola* sp. nov., *A. andra* sp. nov., *A. farrani*, and *A. pallida* are 9.15%, 11.42%, 10.05%, and 13.70% respectively.

#### Amphorina pallida

Taxon classificationAnimalia NudibranchiaEubranchidae

(Alder & Hancock, 1842)

A10B9874-7B1C-5B78-A05F-171F44AA00DE

Figures 1 , 2 , 3 , 4f–g , 7E

Eolis pallida Alder & Hancock, 1842: 35–36.
Eolis minuta Alder & Hancock, 1842: 36.
Eolis picta Alder & Hancock, 1845: fam. 3, pl. 33.
Eolis flavescens Friele & Hansen, 1876: 78.
Eubranchus pallidus (Alder & Hancock, 1842): Edmunds and Kress 1969: 893–896, text figs 1, 3–6.
Amphorina pallida (Alder & Hancock, 1842): Martynov 1998: 775.

##### Material examined.

NE Atlantic, Skagerrak, Sweden, Region Västra Götaland, Bohuslän county, town of Smögen, outermost skerries, Pesaskär (58°35'71"N, 11°18'81"E), 16–30 m depth, 14 Apr 2012, coll. Klas Malmberg (GNM Gastropoda – 8883, two specimens in same lot 10 and 7 mm in length, live, preserved length 7 and 5 mm, respectively). NE Atlantic, Skagerrak, Sweden, Region Västra Götaland, Bohuslän county, town of Smögen, outermost skerries, Pesaskär (58°07'00"N, 10°83'33"E), 10–30 m depth, 01 May 2012, coll. Klas Malmberg (GNM Gastropoda – 8928, four specimens in same lot, 13, 10, 10, and 8 mm in length, respectively, live, preserved length 9,7,7 and 6 mm, respectively). NE Atlantic, the United Kingdom, Scotland, Loch Fyne, Glas Eilean, (56°00'00"N, 05°22'00"W), 16 m depth, 25 Jan 2015, coll. Jim Anderson, one specimen (GNM Gastropoda –9094, preserved length 4 mm). NE Atlantic, Skagerrak, Sweden, Region Västra Götaland, Bohuslän county, town of Smögen, outermost skerries (58°21'00"N, 11°12'00"E), 10–20 m depth, 01 May 2015, coll. Klas Malmberg, four specimens (GNM Gastropoda – 9218, 12 mm in length, live, preserved length 10 mm, GNM Gastropoda – 9219, 14 mm in length, live, preserved length 12 mm, GNM Gastropoda – 9249, 9 mm in length, live, preserved length 7 mm, GNM Gastropoda – 9250, preserved length 3.5 mm). NE Atlantic, the United Kingdom, Scotland, Loch Fyne, Glas, Eilean (55°57'00"N, 05°23'00"W), 16 m depth, 25 Jan 2015, coll. Jim Anderson, one specimen (GNM Gastropoda – 9387, preserved length 10 mm). NE Atlantic, Skagerrak, Sweden, Region Västra Götaland, Bohuslän county, Väderö Islands (58°34'00"N, 11°04'00"E), 20 m depth, 10 Apr 2015, coll. Klas Malmberg, five specimens (GNM Gastropoda – 9443, 10 mm in length, live, preserved length 8 mm, GNM Gastropoda – 9444, 9 mm in length, live, preserved length 7 mm, GNM Gastropoda – 9452, 9 mm in length, live, preserved length 6 mm, GNM Gastropoda – 9453, 7 mm in length, live, preserved length 6 mm, GNM Gastropoda – 9454, 11 mm in length, live, preserved length 9 mm). NE Atlantic, Skagerrak, Sweden, Region Västra Götaland, Bohuslän county, Gullmar Fjord, Släggabåden between Släggö Island, Lysekil and Kristineberg marine station (58°15'70"N, 11°26'60"E), 50–55 m depth, soft clay bottom, 01 Jun 2017, coll. Kennet Lundin (GNM Gastropoda – 9501, 5 mm in length, live, preserved length 4 mm). NE Atlantic, Skagerrak, Sweden, Region Västra Götaland, Bohuslän county, Ide fjord, close to Svarte Jan lighthouse (59°07'00”N, 11°19'00"E), 20 m depth, 01 Sept 2015, coll. Klas Malmberg (GNM Gastropoda – 9695, 4 mm in length, live, preserved length 3 mm). NE Atlantic, the United Kingdom, Northern Ireland, Portaferry (54°23'00"N, 05°35'00"W), 10–20 m depth, soft clay bottom, 14 Mar 2015, coll. Bernard Picton (GNM Gastropoda – 9597, preserved length 5 mm). NE Atlantic, the United Kingdom, Northern Ireland, Portaferry (54°23'00"N, 05°35'00"W), 10–25 m depth, soft clay bottom, 10 Mar 2014, coll. Bernard Picton (GNM Gastropoda – 9601, preserved length 11 mm). NE Atlantic, Norway, Gulen Dive Center (60°57'27.11"N, 5°07'47.10"E), depth 15–20 m, stones, collectors T.A. Korshunova, A.V. Martynov, five specimens (ZMMU Op-708, 17.03.2014, ca. 20 mm in length, live, ca. 8 mm in length, preserved, ZMMU Op-709, 17.03.2014, ca. 15 mm in length, live, ca. 6 mm in length, preserved, ZMMU Op-710, 19.03.2015, 18 mm in length, live, 7 mm in length, preserved, ZMMU Op-711, 07 Mar 2016, 10 mm in length, live, ca. 5 mm in length, preserved, ZMMU Op-712, 12.5 mm in length, live, ca. 6 mm in length, preserved).

##### Diagnosis.

Body up to 25 mm; dorsal pigment spots (if present), small and often rounded, forming an almost continuous orange-brownish covering; in specimens with dorsal pigment spots there is never any colouration of the tail; completely pale specimens likewise lack a tail spot; absence of light pinkish subapical ring on cerata; absence of punctuated white line on external edge of foot; cerata commonly moderate in width without distinctly attenuated apices; digestive gland in cerata relatively broad without distinct short branches; up to four anterior rows of cerata; radular formula 18–41 × 1.1.1, copulative stylet long and bent at the top, receptaculum seminis oval without stalk and widened base.

##### Description.

***External morphology*.** The length of adult specimens may reach 25 mm. The body is narrow. The rhinophores are smooth and 1.5–2 times longer than the oral tentacles. The cerata are relatively long, very broad, with distinctly attenuated apices. Ceratal formula of the specimen ZMMU Op-708 from Norway: right (2, 4, 3, 5; anus, 5, 3, 3, 2, 2) left (1, 3, 3, 5; anus, 4, 4, 3, 2, 2). The foot is narrow, anteriorly without foot corners.

***Colour*.** There are three main and eight subdivisions of colour variations (Fig. 3), from a completely pale body and cerata without spots to specimens with many small rounded reddish orange/brownish pigment spots on the body and cerata. In specimens with distinct dorsal pigment spots, there is never any pigment on the tail. No specimens with blackish body pigmentation or uniformly bright orange colouration were ever observed. Absence of light pinkish subapical ring on cerata. Absence of line or row of dots of white pigment on external edge of foot. The upper part of the rhinophores is often covered with white, relatively dense spots, or in some specimens with dense orange-reddish pigment, but without the formation of pigment rings. The oral tentacles are similarly coloured.

***Anatomy*.** Digestive system (Fig. 4, f3, g3–g7). The jaws are triangularly ovoid. The masticatory processes of the jaws bear a single row of ca. 25 distinct denticles. The radular formula in two studied specimens from Norway (Op-711, Op-712) is 18–31 × 1.1.1. The radular teeth are yellowish. The central tooth is narrow, with a low cusp and four or five lateral denticles (three or less on the anteriormost eroded teeth), including smaller intercalated denticles that may occur in different parts of the tooth.

***Reproductive system*.** (Fig. 7E). The ampulla is relatively small, not distinctly swollen (Fig. 7E, am). The prostate is distinct, extremely long and wide (Fig. 7E, pr). The very large, S-shaped prostate transits to a penial sheath, which contains a conical penis with a long chitinous stylet, bent in the middle (Fig. 4, f4). A supplementary (“penial”) gland is relatively long but thin and inserts into the base of the penis (Fig. 7E, pg). The seminal receptacle is oval (Fig. 7E, rs) without either a stalk or a wide base. The female gland mass includes mucous and capsular glands (Fig. 7E, fgm).

##### Distribution and habitats.

Western Mediterranean Sea and all European Atlantic coasts to northern Norway. On the Swedish west coast, it lives below the halocline.

##### Remarks.

Morphologically *A. pallida* differs from *A. farrani*, *A. viriola* sp. nov., *A. andra* sp. nov., and *A. linensis* by small rounded brownish orange pigment spots on the body (in spotted forms), by small brownish orange spots on the cerata, and by a very large S-shaped prostate.

Minimum uncorrected p-distances of the COI marker which separate *A. pallida* from *A. viriola* sp. nov., *A. andra* sp. nov., *A. linensis*, and *A. farrani* are 8.92%, 9.59%, 10.05%, and 14.31% respectively.

## Discussion

### The nudibranch genus *Amphorina* as a model for ontogenetic periodicity

The genus *Amphorina* is a suitable model for studying the link between a “static” taxonomic system and the underlying evolutionary processes fuelled by ontogenetic periodicity due to both the morphological uniformity across the genus (especially regarding internal characters) on the one hand, and to the large degree of variation in external colouration on the other. Using morphological and molecular data, we show that the genus *Amphorina* is a well-delineated monophyletic genus of the family Eubranchidae (Fig. 1). The validity of the narrowly defined, monophyletic genus *Amphorina*, which was previously resurrected by Martynov (1998), is thus confirmed. The species composition of the genus is restricted here to only five European species (*A. farrani*, *A. viriola* sp. nov., *A. andra* sp. nov., *A. linensis*, and *A. pallida*, Figs 1–7), and a review of this genus is presented for the first time. The genus *Amphorina* is characterised by the presence of up to six anterior ceratal rows, a distinct typically long prostate and a single chitinous penial stylet. The genus *Eubranchus* sensu stricto (type species *E. tricolor* Forbes, 1838) differs considerably from the genus *Amphorina* by the presence of numerous branched ceratal rows, a supplementary gland that is inserted into the vas deferens instead of the penis, the absence of a distinct prostate, and an unarmed penis (Martynov 1998); other eubranchid species are pending review. By the above-listed combination of characters, the genus *Eubranchus* is, in a narrow sense, similar to several other aeolidacean families (see Korshunova et al 2017a, b), but differs from the genus *Amphorina*. The previous unification of the genus *Amphorina* with the genus *Eubranchus* ignored these morphological differences and plainly followed a previous lumping paradigm in nudibranch taxonomy, which has recently been contested (Korshunova et al. 2019b). The concept of multilevel organism diversity (Korshunova et al. 2017a, 2019a) promotes the establishment of small taxonomic units in order to coherently describe hidden diversity at different levels of evolutionary differentiation.

### Ongoing speciation within the *Amphorina* complex in the Skagerrak area

*Amphorina viriola* sp. nov. and *A. andra* sp. nov. are clearly distinguished, with high support by the BPP analysis and also by differences according to the haplogram (Fig. 2A), but the latter also shows a reticulated pattern for *Amphorina andra* sp. nov., which is of relevance for the long-standing problem of speciation. *Amphorina viriola* sp. nov. is apparently at a late stage of the speciation process, since according to ecological, morphological, and genetic data *A. viriola* sp. nov. is separated from *A. andra* sp. nov. but still retains some genetic connection with it. There is a possible window for cross-breeding and subsequent gene flow during periods of storms or upwelling when the surface layer above the halocline temporarily attains a higher salinity, rendering it available for *A. andra* sp. nov. Any species, while forming, must pass through this “reticulated phase” of genetic exchange (e.g., Hennig 1966; Crawford et al. 2015) when it still retains some partial connection with its ancestral species; thus, this case is not only of particular taxonomic interest, but of general evolutionary importance. Recently, multiple evidence was obtained for a very recent speciation event when closely related species formed a reticulated pattern (Burress et al. 2018). Here we show that two *Amphorina* species, *A. viriola* sp. nov. and *A. andra* sp. nov., show significant divergence according to the BPP analysis, demonstrate a statistically well-supported (*p* = 0.007, Fig. 2B) difference in ecological niches/environment (including robust bathymetrical differences correlated with drastic salinity differences, characteristic for the marine waters of southwestern Sweden), also possess minor morphological differences, and at the same time form a partly reticulated pattern according to the molecular phylogenetic data (Fig. 1). This is in line with proposals that coalescent analysis should be supplied together with phenotypic and ecological data (Sukumaran and Knowles 2017).

The present case clearly differs from the situation when a reticulated molecular phylogenetic pattern of two closely related species was used for evidence of their synonymy (Ludt et al. 2019), because significant molecular and ecological data are presented for two *Amphorina* species and their ongoing speciation processes. When a species is still in the process of speciation (and we can expect it for a majority of species) it must preserve various degrees of connection with an ancestral species (an ancestral group of populations) and hence some ability to hybridise with the ancestral species. Such processes will lead to a partially reticulated pattern of the obtained phylogenetic trees. There are also previous data that taxonomically recognised species and genera, from invertebrates to hominins, are able to hybridise with fertile offspring. Therefore, there is no contradiction when species with a significant degree of incomplete speciation show some reticulated phylogenetic patterns and sometimes very insignificant genetic differences within taxonomically recognised species. One of the most evident cases is the innumerable African cichlid species (Koblmüller et al. 2019), for many of these have very low genetic divergences (0.1–0.25%) and evidence has repeatedly shown a substantial gene flow among numerous taxonomically well-established species (Burress et al. 2018; Gante et al. 2016; Malinsky et al. 2018). This pattern is very similar to what we found here for two *Amphorina* species. In support of the model presented here, there is evidence that a brackish-water environment, and particularly the waters in the eastern Skagerrak, Kattegat, and Baltic regions, strongly facilitates the formation of new organism groups/units that can be taxonomically evaluated from genus (Korshunova et al. 2018), to species (Momigliano et al. 2017) or to a specific population (Berg et al. 2015).

Remarkably, both species, *A. viriola* sp. nov. and *A. andra* sp. nov., occur in the same geographical region on the coast of southwestern Sweden, which is characterised by the presence of two different bathymetric layers, one that corresponds to the Baltic-influenced brackish surface layer, where *A. viriola* sp. nov. is found, whereas the deeper layer represents close to normal oceanic salinity. The Kattegat area between Sweden and Danish Jutland receives brackish water from the Baltic Sea via the Bälten and Öresund straits in the south and the so-called Baltic surface current flows onward north along the Swedish west coast. The difference in salinity leads to a distinct halocline in the Kattegat and the eastern part of the Skagerrak, at ca. 15 metres depth, with a layering of brackish surface water and saltier deep-water. The western part of Skagerrak has no such layering, and here the salinity is high from the surface to the bottom. At the southernmost part of the Kattegat the salinity of the surface layer is only ca. 8‰ but increases successively northward. At the Swedish coast of the Skagerrak, the salinity of the surface layer is usually approximately 24–25‰, but it is highly variable with extremes ranging from 12 to 30‰ depending on weather conditions and strong winds. The deep-water layer below the halocline is, by contrast, much more stable in salinity, with 32–34‰. In the Gullmar fjord and the Ide fjord the halocline is shallower than 15 metres, usually ca. 6–7 metres, and there is an outflow of freshwater from river outfalls along the inner parts of the fjords. There is also freshwater outflow to the Swedish west coast from the two largest rivers in the area, the Göta river, entering at the port of Gothenburg, and Glomma river entering the Oslo fjord in Norway. The latter has a large seasonal impact on the northernmost part of the Swedish coast of Skagerrak, especially in spring, during snow melt in the mountains. Another factor in maintaining a long-term stability of bathymetric layers is the very low tidal exchange in the area, normally only 20 cm in Skagerrak. In this study we performed a statistical test for the bathymetric distribution of the two species *A. viriola* sp. nov. and *A. andra* sp. nov. and confirmed with high support (*p* = 0.007) that *A. viriola* sp. nov. and *A. andra* sp. nov. are very strictly divided, according to the brackish water and oceanic salinity layers (halocline) without any overlap (Fig. 2B). Thus, these results robustly confirm firm the ecological differentiation between these two species.

Taking into consideration the population-to-species continuum (Coates et al. 2018) and the artificial strict distinction of species for taxonomic purposes (Zachos 2018b), we cannot evaluate the group of nudibranchs presented here as simply a modified population since it shows stable morphological, genetic and ecological features. The current system of zoological nomenclature was formed during pre-evolutionary times. It does not address the underlying genetic-epigenetic processes and provides only a very rigid application of a name to some “type specimen”. At the same time, an arsenal of various molecular, phylogenetic and delimitations methods that can detect subtle, but statistically reliable, differences between organism groups are in direct contradiction with the persistent system of nomenclature. Therefore, under the putatively same “species rank” various natural organism entities/groups, at a very different degree of a very complex population to species continuum (Coates et al. 2018), can be concealed if the evolutionary processes in the current nomenclature system are insufficiently estimated. The case of the small genus *Amphorina* clearly demonstrates such multilevel organism diversity (Korshunova et al. 2019a) at different stages of speciation/evolutionary differentiation. Notably, all species show similar external and internal traits, which can be easily confused even by an expert not specifically trained for that genus. But according to the integrative data for these species, *A. pallida* definitely has a stronger degree of differentiation from other *Amphorina* species than the differentiation between *A. farrani* and *A. andra* sp. nov. In turn, *A. andra* sp. nov., has a much lesser degree of differentiation from *A. viriola* sp. nov. than the latter does from *A. pallida*. However, despite that the degree of “speciation” is different in all these organism groups, they are still considered to fall within the “species category”. Notably, *Amphorina viriola* sp. nov. shows a similar periodic-like pattern of different colouration morphs as its sister species, the closely related *A. andra* sp. nov. and *A. farrani*. (Fig. 3). Similar periodic patterns in colouration are demonstrated in all five species of the genus *Amphorina*, together constituting a “species complex” that is difficult to distinguish, while at the same time it provides a model for the investigation of periodic morphological patterns for taxonomy. The data presented in this study thus allow for integrating robust evidence of speciation, from an evolutionarily little assessed invertebrate group, with the most current and important topic of periodic patterns in the formation of morphological diversity (Haupaix and Manceau 2018).

### Periodic patterns in organism diversity facilitate fine-scale species delimitation: the nudibranch case

Periodic-like patterns in application to biology, though discussed for a long time (e.g., Vavilov 1922; Hess 2000), and successfully applied for protein structure (Taylor 2002), have only recently been proposed for applied use in taxonomy and phylogeny (Martynov and Korshunova 2015). In this example, evident periodicity was revealed for a higher-level organism group using an ontogenetic phylotypic stages approach. Indeed, compared to the stricter periodic system in chemistry, variability of biological organisms extends far beyond those of regular parallel rows (e.g., Bolnik et al. 2019). However, there are many examples when various features appear parallel in related taxa. For instance, in the present study we confirmed a remarkable parallelism in the colouration of several separate, but related, species of the genus *Amphorina*. Recently, interest in periodic patterns in biology was reviewed and several studies found evident periodic patterns of colouration in birds and other vertebrate groups, and also found a direct link to constraints in early developmental patterns (Haupaix et al. 2018, Haupaix and Manceau 2019). Thus, the idea was further confirmed that periodic patterns in adult morphology of different taxa are underlined by early developmental factors. Therefore, even in a majority of other cases where we do not have data on early development, we can reasonably infer that ontogenetic periodicity must influence adult morphology in the majority of metazoans, since all of them possess a similar homeobox system of early development (Holland 2013). For example, colour and pattern polymorphism of land snail shells of the genus *Cepaea* has been shown to be caused by a complex interaction between gene expression and local environment, with both random and regular colour patterns (e.g., Jones et al. 1977; Cook 2017; Davison et al. 2019). The underlying genetic basis for the appearance of any characters can thus be either very complex or simple and irregular, but when such variations are brought up to higher taxonomic and phylogenetic levels, the periodic/quasi-periodic patterns become more evident, although still with irregularities. For example, the helicid land snails turned out to be a polyphyletic assemblage, but they share a similar degree of polymorphism in parallel in several lineages (Neiber and Hausdorf 2015). Our approach implies potential analysis within a periodic framework of any of the characters, not only of colour, which emphasises the interspecific periodic patterns rather than intraspecific, more continuous variations. Therefore, the evaluation of periodic patterns in external appearance is a useful tool for identification in cases where species are difficult to delimit. The present *Amphorina* case is a suitable example because it comprises several very closely related species, all of them demonstrating similar patterns of genetic variability (Figs 1, 3), and at the same time it includes an evident example of a late stage of speciation. All these factors make species delimitation using traditional taxonomic or standard modern approaches particularly difficult.

The appearance of similar colour patterns across different species of the genus *Amphorina* can reasonably be termed periodic patterns, although this periodicity indeed only partly approaches the periodicity which is known in chemistry (Babaev and Hefferlin 1996), with considerable reservations. In biology, the main problem of the justification of periodicity is that among numerous characters it is possible to arbitrarily choose some that fit periodic patterns (Popov 2002; Babaev 2019). In the present case, however, we detected that colour periodicity is a part of natural polymorphism within a molecularly proven group (Fig. 1) of closely related species in the genus *Amphorina*. These similar colour variations appear in parallel, periodically, within the different species and immediately influence the key features for taxonomic diagnoses and cannot be discarded as auxiliary characters. This allows the investigation of periodic patterns in similar phylotypic periods to continue among distantly related families within higher-ranked monophyletic taxonomic groups (Martynov and Korshunova 2015). Since colour polymorphism is influenced by some periodicity at the level of the developmental genes it can be used as an underlying source of periodic patterns in biodiversity and systematics. Thus, the vertical columns represent particular species, whereas horizontal periods are patterns of colouration within the genus *Amphorina*. For each species within that genus a periodic appearance of a similar colour pattern can be expected (Fig. 3).

Application of a periodic-like arrangement of vertical rows and horizontal periods helps to highlight subtle differences between apparently highly similar forms. For example, some white forms with distinct yellow-orange spots of *A. farrani* are very similar to corresponding forms of *A. andra* sp. nov., but in the latter, a distinct yellow-orange pigment spot or stripe on the tail is commonly absent (Fig. 3). Such a character is very easy to overlook in the traditional “overall differences” approach, whereas a periodic-like arrangement makes it evident. Furthermore, by using a periodic approach in taxonomy we can detect the absence of some particular colour forms in closely related species, thus revealing its predictive function, as is common in chemistry. For example, uniformly coloured bright orange specimens were discovered for *A. andra* sp. nov., but not for the closely related *A. viriola* sp. nov. or *A. farrani*, despite the investigation of hundreds of specimens (Fig. 3). Either such a morph for some reason does not exist in *A. viriola* sp. nov. or *A. farrani*, or it can potentially be discovered in the future. Perhaps a more instructive example is when forms with dark surface pigmentation do occur within both *A. viriola* sp. nov. and *A. andra* sp. nov., but are not yet known in *A. farrani* (Fig. 3). Because there are forms with dark underlying body colour within *A. farrani* (Fig. 3) it is reasonable to expect future findings of forms with extensive dark surface pigmentation also within *A. farrani*. Such predictive functionality thus facilitates species delimitation but also can compel taxonomy to become a more rigorous discipline, along with molecular phylogeny, and prevent simply chaotically mapping morphological features within apparently “indistinguishable species complexes”. The periodic approach to biological taxonomy coupled with molecular analysis has the potential for various organismal groups, not only molluscs, because when arranging all the particular character states/colour patterns detected for some particular species, it is easier to distinguish species complexes, by a process of identifying successively finer details. This is useful for various practitioners, especially for citizen scientists, not as an artificial addition to already established taxonomic methods, but rather as a mapping of naturally existing patterns of biological diversity. Finer analysis shows that such complexes are possible to distinguish morphologically, by using a combination of various methods including the periodic approach suggested here. Several further studies on different groups, such as rodents (Johnson et al. 2018) and fishes (Gante 2018; Salis et al 2019), confirmed the existence of periodic patterns during the development of morphological characters, yet without direct construction of periodic-like tables, which can be a next step. Thus, these complex periodic-like genetic-epigenetic interactions within an ontogenetic framework can work as a theoretical foundation and confirmation of the practical validity of a periodic approach in taxonomy and phylogeny.

## Supplementary Material

XML Treatment for Amphorina

XML Treatment for Amphorina farrani

XML Treatment for Amphorina viriola

XML Treatment for Amphorina andra

XML Treatment for Amphorina linensis

XML Treatment for Amphorina pallida

## Supplementary materials

Supplementary material 1 Table S1. List of samples, localities, GenBank accession numbers, and voucher references Data type: species data https://binary.pensoft.net/file/387257This dataset is made available under the Open Database License (http://opendatacommons.org/licenses/odbl/1.0/). The Open Database License (ODbL) is a license agreement intended to allow users to freely share, modify, and use this Dataset while maintaining this same freedom for others, provided that the original source and author(s) are credited.Tatiana Korshunova, Klas Malmberg, Jakov Prkić, Alen Petani, Karin Fletcher, Kennet Lundin, Alexander Martynov
