# Supplementary material for: Fine-scale species delimitation: speciation in process and periodic patterns in nudibranch diversity
Source: Zookeys. 2020 Mar 9;917:15–50. doi: 10.3897/zookeys.917.47444 (PMC7076062; doi:10.3897/zookeys.917.47444)
Supplement: Supplementary material 1 [file zookeys-917-015-s001.doc]

Table S1. List of specimens used for the molecular analyses.

| **Species** | **Locality** | **Registration** | **BPP**  **primary**  **division** | **GenBank accession no.** | | |
| --- | --- | --- | --- | --- | --- | --- |
|  |  |  |  | **COI** | **16S** | **H3** |
| *Amphorina* *viriola* sp. nov. | Sweden: Idefjord  brackish water | GNM9393 | A | **MN868697** | **MN865239** | **MN862678** |
| *Amphorina* *viriola* sp. nov. | Sweden: Idefjord  brackish water | GNM9394 | A | - | **MN865240** | **MN862679** |
| *Amphorina* *viriola* sp. nov. | Sweden: Idefjord  brackish water | GNM9395 | A | - | **MN865241** | **MN862680** |
| *Amphorina* *viriola* sp. nov. | Sweden: Idefjord  brackish water | GNM9396 | A | - | **MN865242** | **MN862681** |
| *Amphorina* *viriola* sp. nov. | Sweden: Idefjord  brackish water | GNM9397 | A | **MN868698** | - | **MN862682** |
| *Amphorina* *viriola* sp. nov. | Sweden: Idefjord  brackish water | GNM9398 | A | - | **MN865243** | **MN862683** |
| *Amphorina* *viriola* sp. nov. | Sweden: Idefjord  brackish water | GNM9936 | A | **MN868678** | **MN865222** | **MN862684** |
| *Amphorina* *viriola* sp. nov. | Sweden: Lysekil  brackish water | GNM9260 | B | KY513660 | KY513638 | - |
| *Amphorina* *viriola* sp. nov. | Sweden: Lysekil  brackish water | GNM9261 | B | KY513661 | KY513639 | KY513681 |
| *Amphorina* *viriola* sp. nov. | Sweden: Lysekil  brackish water | GNM9262 | B | **MN868680** | **MN865224** | **MN862673** |
| *Amphorina* *viriola* sp. nov. | Sweden: Lysekil  brackish water | GNM9263 | B | KY513662 | KY513640 | - |
| *Amphorina* *viriola* sp. nov. | Sweden: Lysekil  brackish water | GNM9264 | B | KY513663 | KY513641 | KY513682 |
| *Amphorina* *viriola* sp. nov. | Sweden: Lysekil  brackish water | GNM9265 | B | KY513664 | KY513642 | KY513683 |
| *Amphorina* *viriola* sp. nov. | Sweden: Lysekil  brackish water | GNM9093 | B | - | KY513653 | KY128614 |
| *Amphorina* *viriola* sp. nov. | Sweden: Smögen  brackish water | GNM9341 | B | **MN868681** | **MN865225** | **MN862675** |
| *Amphorina* *viriola* sp. nov. | Sweden: Smögen  brackish water | GNM9360 | B | **MN868696** | **MN865238** | **MN862676** |
| *Amphorina* *andra* sp. nov. | Norway | ZMMU: Op-701 | C | **MN868677** | **MN865221** | **MN862685** |
| *Amphorina* *andra* sp. nov. | Croatia | ZMMU: Op-703 | C | **MN868679** | **MN865223** | **MN862686** |
| *Amphorina* *andra* sp. nov. | UK | GNM9272 | C | KY513670 | KY513648 | KY513689 |
| *Amphorina* *andra* sp. nov. | Italy | GNM9292 | C | KY513678 | KY513657 | - |
| *Amphorina* *andra* sp. nov. | Italy | GNM9293 | C | KY513679 | KY513658 | KY513699 |
| *Amphorina* *andra* sp. nov. | Spain | GNM9295 | C | KY513675 | KY513654 | - |
| *Amphorina* *andra* sp. nov. | UK | GNM9266 | C | KY513665 | KY513643 | KY513684 |
| *Amphorina* *andra* sp. nov. | Sweden: Smögen | GNM9716 | D | **MN868695** | **MN865237** | - |
| *Amphorina* *andra* sp. nov. | Sweden: Smögen | GNM9717 | D | **MN868693** | **MN865235** | - |
| *Amphorina* *andra* sp. nov. | Sweden: Smögen | GNM9720 | D | **MN868694** | **MN865236** | - |
| *Amphorina farrani*  (Alder & Hancock, 1844) | UK | GNM9268 | F | **MN868687** | **MN865229** | **MN862674** |
| *Amphorina farrani*  (Alder & Hancock, 1844) | Spain | ZMMU: Op-704 | F | **MN868685** | **MN865228** | **MN862687** |
| *Amphorina farrani*  (Alder & Hancock, 1844) | Spain | ZMMU: Op-705 | F | **MN868686** | - | **MN862688** |
| *Amphorina farrani*  (Alder & Hancock, 1844) | UK | GNM9267 | F | KY513666 | KY513644 | KY513685 |
| *Amphorina farrani*  (Alder & Hancock, 1844) | UK | GNM9269 | F | KY513668 | KY513646 | KY513687 |
| *Amphorina farrani*  (Alder & Hancock, 1844) | UK | GNM9270 | F | KY513669 | KY513647 | KY513688 |
| *Amphorina farrani*  (Alder & Hancock, 1844) | UK | GNM9273 | F | KY513671 | KY513649 | KY513690 |
| *Amphorina farrani*  (Alder & Hancock, 1844) | France | GNM9278 | F | KY513672 | KY513651 | KY513693 |
| *Amphorina farrani*  (Alder & Hancock, 1844) | France | GNM9279 | F | KY513673 | KY513652 | KY513694 |
| *Amphorina farrani*  (Alder & Hancock, 1844) | Spain | GNM9296 | F | KY513676 | KY513655 | KY513696 |
| *Amphorina farrani*  (Alder & Hancock, 1844) | Spain | GNM9298 | F | KY513677 | - | KY513698 |
| *Amphorina linensis*  (Garcia-Gomez, Cervera & Garcia, 1990) | Croatia | ZMMU: Op-706 | L | **MN868682** | **MN865226** | **MN862689** |
| *Amphorina linensis*  (Garcia-Gomez, Cervera & Garcia, 1990) | Croatia | ZMMU: Op-707 | L | **MN868683** | **MN865227** | - |
| *Amphorina linensis*  (Garcia-Gomez, Cervera & Garcia, 1990) | Sweden: Väderöarna | GNM9392 | L | **MN868684** | - | **MN862677** |
| *Amphorina pallida*  (Alder & Hancock, 1842) | Norway | ZMMU: Op-708 | P | **MN868688** | **MN865230** | **MN862690** |
| *Amphorina pallida*  (Alder & Hancock, 1842) | Norway | ZMMU: Op-709 | P | **MN868689** | **MN865231** | **MN862691** |
| *Amphorina pallida*  (Alder & Hancock, 1842) | Norway | ZMMU: Op-710 | P | **MN868690** | **MN865232** | **MN862692** |
| *Amphorina pallida*  (Alder & Hancock, 1842) | Norway | ZMMU: Op-711 | P | **MN868691** | **MN865233** | **MN862693** |
| *Amphorina pallida*  (Alder & Hancock, 1842) | Norway | ZMMU: Op-712 | P | **MN868692** | **MN865234** | **MN862694** |
| *Amphorina pallida*  (Alder & Hancock, 1842) | UK | GNM9094 | P | KY129030 | KY128821 | KY128616 |
| *Eubranchus tricolor*  Forbes, 1838 | Norway | ZMMU: Op-525 | O | MF523379 | MF523399 | MF523304 |
| *Eubranchus tricolor*  Forbes, 1838 | Sweden: Smögen | GNM9096 | O | KY129032 | KY128823 | KY128618 |
